# First osteohistological and histotaphonomic approach of *Equus occidentalis* Leidy, 1865 (Mammalia, Equidae) from the late Pleistocene of Rancho La Brea (California, USA)

**DOI:** 10.1371/journal.pone.0261915

**Published:** 2021-12-28

**Authors:** Rodrigo Leandro Tomassini, María Dolores Pesquero, Mariana Carolina Garrone, María Dolores Marin-Monfort, Ignacio Alejandro Cerda, José Luis Prado, Claudia Inés Montalvo, Yolanda Fernández-Jalvo, María Teresa Alberdi

**Affiliations:** 1 INGEOSUR, Departamento de Geología Universidad Nacional del Sur (UNS)-CONICET, Buenos Aires, Argentina; 2 Departamento de Paleobiología, Museo Nacional de Ciencias Naturales (CSIC), Madrid, Spain; 3 Departamento de Botánica y Geología, Universidad de Valencia, Burjassot, Valencia, Spain; 4 CONICET-Instituto de Investigación en Paleobiología y Geología, Museo Carlos Ameghino, Universidad Nacional de Río Negro, Cipolletti, Río Negro, Argentina; 5 INCUAPA-Departamento de Arqueología, Universidad Nacional del Centro, Olavarría, Buenos Aires, Argentina; 6 Facultad de Ciencias Exactas y Naturales, Universidad Nacional de La Pampa, Santa Rosa, La Pampa, Argentina; Griffith University, AUSTRALIA

## Abstract

Rancho La Brea (California, USA) is the most emblematic Quaternary fossiliferous locality in the world, since both the high number and diversity of the specimens recovered and their excellent preservational quality. In the last decades, paleobiological and paleoecological knowledge of the different groups of mammals from this site has increased notably; however, some aspects have not yet been inquired or there is little information. In this work we provide information on one of the most abundant mammals of this site, the equid *Equus occidentalis*, based on the study, from osteohistological and histotaphonomic perspectives, of thin sections of different limb bones. On the one hand, from an osteohistological viewpoint, we observe that the distribution and characterization of bone tissues in the different skeletal elements are, in general lines, similar to that mentioned for other extant and extinct equids. Cyclical growth marks allowed us to propose preliminary skeletochronological interpretations. On the other hand, from a taphonomic viewpoint, we note that all the samples reflect an excellent preservation of the bone microstructure, slightly altered by different pre- and post-burial processes. The variations recorded evidence different taphonomic history and preservation conditions among pits. This is the first study including fossil material from Rancho La Brea exclusively based on the analysis of the bone microstructure features.

## Introduction

Rancho La Brea (Los Angeles, California, USA) comprises several asphalt seeps corresponding to emanations of the Salt Lake Oilfield. According to the values of obtained ^14^C dating, the age of the fossils from these deposits ranges from more than 50,000 to less than 10,000 years (e.g., [1–4]). The high number of specimens (>3.5 million specimens representing >600 species) recovered, including vertebrates (mammals, birds, reptiles, amphibians, and fishes), invertebrates, plants, and coprolites, and their excellent preservational quality, make Rancho La Brea site a true *lagerstätte* and offer unsurpassed insights into a past ecosystem of the last ice age (e.g., [3, 5–8]). These features make Rancho La Brea one of the most emblematic Quaternary fossiliferous locality in the world.

It is known that natural asphalt can be extremely sticky and that seeps of only a few centimeters in depth suffice to immobilize large domestic animals such as cows and horses [9]. It was proposed and widely accepted that, during the late Pleistocene, the open asphalt seeps of Rancho La Brea site would have acted as episodic traps for thousands of vertebrates (e.g., [5, 8–11]). In this unusual taphonomic context, the fossil record of vertebrates consists of columns of jumbled bone remains mostly incomplete and disarticulated, mixed together with little stratigraphic order [2, 12].

Despite the importance of the Rancho La Brea site, well-known as a source for bitumen by Native Americans for thousands of years, also known by the European settlers and shown as an exceptional fossiliferous site already in 1875, systematic excavations did not take place until the 20th century and no dates were made until the 21st century [4]. In the case of vertebrates, most scientific studies are focused mainly on taxonomic, osteological, paleopathological, and isotopic analyses. However, research on the taphonomy of the assemblages are scarce (e.g., [2, 5, 13]) and limited to only a few pits (see [14]), so that several questions to understand how the site formed remain unresolved and other possible scenarios should not be ruled out.

Even though morphological analysis of fossil bones has constituted the principal source of information in vertebrate paleobiology (e.g., [15]) and paleoecology (e.g., [16, 17]), other approaches can provide complementary data. Osteohistology is an important tool to evaluate biological issues of extinct taxa (e.g., [18–24]); however, in the case of mammals, there are still a large number of taxa that have not been addressed in detail. Particularly for equids, in the last decades the analysis of bone microstructure (e.g., type of matrix, degree of vascularization, presence of growth marks) was applied to infer different life history traits of the individuals, such as longevity, body size, growth rates, sexual dimorphism, maturity age, and soft tissue reconstruction, among others, of both extant and extinct representatives (e.g., [25–28]). Taking into account that diverse post-mortem events, both before and after the burial (e.g., death history, decomposition trajectory, depositing environment itself), can produce modifications that directly affect the original microstructural features, it is also important to evaluate the preservation features of the bone histology (e.g., [29–32]); this is the basis of what has been re-named as histotaphonomy [33].

We study here, from different perspectives, thin sections of *Equus occidentalis* Leidy [34] (Mammalia, Equidae) limb bones (humerus, radius, femur, metacarpal, and metatarsal) recovered from late Pleistocene tar pits of Rancho La Brea site. The selection of this species is based on the fact that it represents one of the most abundant herbivores on Rancho La Brea site [35, 36] and there are several works on other species of horses, both extant and extinct, that allow us to propose a detailed comparative framework. We analyze the osteohistological features to establish: 1) growth patterns; 2) ontogenetic stages; and 3) estimated death ages of the individuals. Also, we evaluate different post-mortem processes that modified the original bone microstructure to reconstruct the possible taphonomic histories of the remains and the environmental context of preservation. This paper is a preliminary multiproxy approach on different living and post-mortem events that yields general information on the paleobiology and paleoecology of this species and on the formation of the fossil-bearing levels.

## Material and methods

### Specimens studied

All the specimens of *E*. *occidentalis* here studied are hosted in La Brea Tar Pits and Museum, George C. Page Museum collection, a branch of the Natural History Museum of Los Angeles (Los Angeles, California, USA), under the acronyms LACMHC and Z. Taking into account that the proposed analysis involves a destructive methodology, for this preliminary study we could make thin sections only of two humeri (LACMHC 25297 and LACMHC 25346), one radius (LACMHC 6154), one femur (LACMHC 27421), two III metatarsals (Mt-III; Z 4657 and Z 4697), and two III metacarpals (Mc-III; LACMHC 26263 and LACMHC 26267) (Table 1). All necessary permits were obtained for the described study, which complied with all relevant regulations. The permit (NHMLAC Outgoing Loan Number: 17221) to make these analyses was awarded by the Natural History Museum to one of us (J.L.P).

**Table 1:** List of the *Equus occidentalis* specimens used in this study, general information of the materials, and provenance data.

Specimen	Element	Preserved portion	Thin section	Provenance	Depth (cm)
LACMHC 25297	Left humerus	Distal	Distal level	Tar pit 61	259.08–304.8
LACMHC 25346	Right humerus	Distal	Distal level	Tar pit 77	274.32–335.28
LACMHC 6154	Left radius	Distal	Distal level	-	-
LACMHC 27421	Left femur	Mid-shaft and distal	Mid-shaft level	Tar pit 60 (grid D12)	396.24–426.72
LACMHC 26267	Right Mc-III	Proximal	Proximal level	Tar pit 77 (grid F11)	365.76–457.2
LACMHC 26263	Right Mc-III	Mid-shaft and distal	Mid-shaft level	Tar pit 67 (grid H11)	518.16–579.12
Z 4697	Left Mt-III	Proximal and mid-shaft	Mid-shaft level	Tar pit 77 (grid E9)	320.04–381
Z 4657	Right Mt-III	Proximal and mid-shaft	Mid-shaft level	Tar pit 67	-

Specimens come from different pits, including 60, 61, 67, and 77. There is no data on the pit of radius LACMHC 6154. All the specimens were broken and incomplete. Based on the data of the fossiliferous levels (grid and depth; see Table 1), it cannot be confirmed that specimens coming from the same pit (i.e., 67 and 77) correspond to a single individual. Pits involved spanning the latest Pleistocene, from ~35 ka to 11.5 ka ([3]; see also [37]). It is important to note that, in several cases, fossil bones from a single pit show important age variations (see [37]). In this sense, a recurrent problem in this site is the assignment of average ages for undated fossils of a recovered from a same pit (“pit averaging” see [8, 37]).

Specimens were photographed before cutting. They were transversely cut, obtaining a sample of ~3 cm thick. The cuts were made in different sectors according to the preserved portion of each specimen (Table 1, S1 Fig); we considered this aspect when making comparisons with other taxa. We performed a complete mold and cast of the cut portions to avoid the loss of anatomical information for future studies (see [38]). The sampling was made by Gary Takeuchi (Collections Manager at La Brea Tar Pits and Museum), following the protocol established in Lamm [39].

### Preparation of histological thin sections

The bone microstructure of the different elements is evaluated based on transverse thin sections. The sections were made at the Laboratorio de Petrotomía of the INGEOSUR, Departamento de Geología, Universidad Nacional del Sur-CONICET (Bahía Blanca, Argentina). All the specimens are broken; the medullary cavity of LACMHC 25346, LACMHC 26267, Z 4657, LACMHC 27421, and LACMHC 25297, is completely filled with sediments impregnated with asphalt.

We had different problems during the elaboration of the thin section following standard techniques (e.g., [40]), because hydrocarbons act as a release agent, which causes the resins commonly used for gluing or embedding not to adhere to the fossil remains (see [41]). To avoid this setback, in this case, the chips (= blocks) were made embedding the samples in a large volume of low-viscosity epoxy resin (DICAST 867) and leaving edges higher than 10 mm around the sample. The large volume generates a higher shrinkage, preventing the separation of the chip components (resin/fossil), while the low viscosity allows a higher penetration in the bone tissues. Chips were sectioned with a diamond saw (MK-303 professional), obtaining samples of 1 cm thick, and then ground and polished with silicon carbide (from #320 to #1200 grit) in a grinder machine. Before the mounting, excess resin was removed, in order to reduce the contact surface between resin and glass slide and thus prevent posterior breakages by resin shrinkage. Chips were mounted on the glass slides using UV acrylic resin Bohle 660 under an ultraviolet light lamp, since (unlike epoxy resins) it does not require heat application, optimizes adhesion, and reduces the curing time, which allows cutting and polishing quickly after mounting. Sections of 200 mm thick were obtained using a diamond saw. Finally, sections were ground and polished using a 380 rpm grinding disc and on glass, with different silicon carbide (#320, #600, #1000, and #1200 grit), to a thickness of 100±10 μm. During polishing, it was necessary to reduce the cleaning time with ultrasound to avoid peeling and losing portions of the sample.

### Osteohistological analysis

Analysis and high-resolution imaging of the transverse thin sections have been performed under a Nikon Eclipse E400 POL petrographic microscope, with polarized light, a 1/4λ filter, and an incorporated digital camera, which belong to the Departamento de Geología, Universidad Nacional del Sur (Bahía Blanca, Argentina). Measurements of the samples (see Table 2) were obtained by the use of Image J® [42].

**Table 2:** List of *Equus occidentalis* limb bone samples analyzed and measurements calculated on the thin sections.

Specimen	Skeletal element	Bone área (mm^2^)	Medullary area (mm^2^)	Bone diameter (mm)		Compact cortex thickness (mm)		LAG	EFS
				anterior-posterior	transversal	Mi	Ma		
LACMHC 25346	Humerus	1715.56	418.18	51.99	44.34	8.19	16.48	1	Absent
LACMHC 25297	Humerus	1658.97	376.98	50.39	44.18	8.18	15.20	2	Absent
LACMHC 6154	Radius	1196.52	220.91	29.64	93.96	8.28	14.88	0	Absent
LACMHC 27421	Femur	2029.93	1094.47	66.39	47.42	2.11	24.16	0	Absent
LACMHC 26263	Mc-III	901.47	114.31	28.95	38.91	7.12	13.70	3	Present
LACMHC 26267	MC-III	1009.73	168.39	29.92	41.15	6.22	12.59	3	Absent
Z 4697	Mt-III	1117.77	162.47	34.89	41.04	8.46	13.31	2	Present
Z 4657	Mt-III	1133.19	186.62	34.89	41.04	7.75	14.19	2	Present

Abbreviations: EFS. external fundamental system. LAG. line of arrested growth. Ma. maximum value. Mi. minimum value.

To avoid losing material during the preparation of thin sections, considering the difficulties of the alternative methodology used here (see above), some thin sections were thicker than standard paleohistological sections, obscuring details as the osteocyte lacunae shape. The intrinsic fiber organization based on the optical properties of the bone cannot be assessed in these sections. For this reason, in some instances, the nature of the extracellular matrix is indirectly inferred based on parameters as the vascular pattern. The histological description of bone microstructure follows Francillon-Vieillot et al. [19] and de Ricqlès et al. [43]. For histological characterization, we consider the following parameters: presence and distribution of primary and secondary tissues, vascular pattern, form, density and order of osteocytes lacunae, presence and distribution of Sharpey’s fibers, number and distribution of cyclical growth marks (lines of arrested growth -LAGs- and annuli).

A skeletochronological analyses (age estimation from cyclical growth marks counting) was performed considering the number of LAGs. Assuming that, at least in mammals, LAGs are deposited annually, independently of metabolic rate and climatic background (see [44, 45]), we estimate the age at the moment of death of each specimen based on the total number of LAGs present in the primary cortical bone. We count LAGs that can be traced along the whole section, although, in some cases, it is difficult because they are partially erased by secondary osteons [46].

### Microstructural post-mortem changes

A macroscopic analysis was performed on each specimen before cutting to identify taphonomic surface modifications caused by physical processes, including weathering, abrasion, and breakage. Weathering was evaluated following the six stages scale proposed by Behrensmeyer [47], which ranges from 0 (absent weathering) to 5 (extreme weathering). Abrasion was evaluated using the three stages defined by Alcalá [48]: intact, rounded, and polished remains. Breakage was evaluated considering if the specimens showed biostratinomic (produced in relatively fresh or dry bone) or fossil-diagenetic (produced in recrystallized or permineralized bone) fractures [49].

Chips and thin sections were examined and analyzed, from a taphonomic viewpoint, using a light binocular microscopy Nikon SMZ-1 (magnification × 10–60) and SEM imaging to identify modifications of the bone microstructure. Histological destruction of bone due microbial attack (e.g., bacteria, fungi) was categorized and described according to the Oxford Histology Index (OHI) proposed by Hedges et al. [29], ranging from stage 0 (with no original histological features identifiable other than Haversian canals) to stage 5 (with less than 5% of the microstructure affected). The different types of microscopic focal destruction (MFD) are classified based on their size, shape, and the presence of a hypermineralized ring [50, 51]. The type of microbial alteration was determined using the descriptions provided by Hackett [50] divided in: 1) Wedl MFD with irregular tunnels, branched or not, caused by fungi, and 2) the non-Wedl MFD with linear longitudinal, budded and lamellate tunneling caused by bacteria. A FEI QUANTA 200 and FEI Inspect (Low Vaccum) SEM microscope equipped with an X-ray energy dispersive spectrometer (EDS) was used at the Museo Nacional de Ciencias Naturales (MNCN-CSIC; Madrid, Spain). Analyses were performed in secondary electron emission (SE-SEM) and backscattered electron (BSE-SEM) modes.

The microcracks associated with secondary osteons [52–54] were distinguished according to their position and orientation in the following types: (1) central radial microcracks spreading from the Haversian canal outwards; (2) circumferential cracks around the outer margin of the osteons separating them partially from adjacent structures; (3) peripheral radial cracks spreading from the cement line inwards; and (4) radial microcracks cutting through the cement lines of secondary osteons across the cement lines connecting with adjacent osteons. The fissures larger than cracks, either influenced or non-influenced by the histological structures of bones were also described.

We evaluated the presence of fissures and the tissues affected by them. Finally, the mineral infilling of the microstructural cavities (e.g., canaliculi, osteocyte lacunae, and Haversian and Volkmann canals), medullary cavity, and microcracks and fissures, was determined.

## Results

### Osteohistology

**Fig 1:**
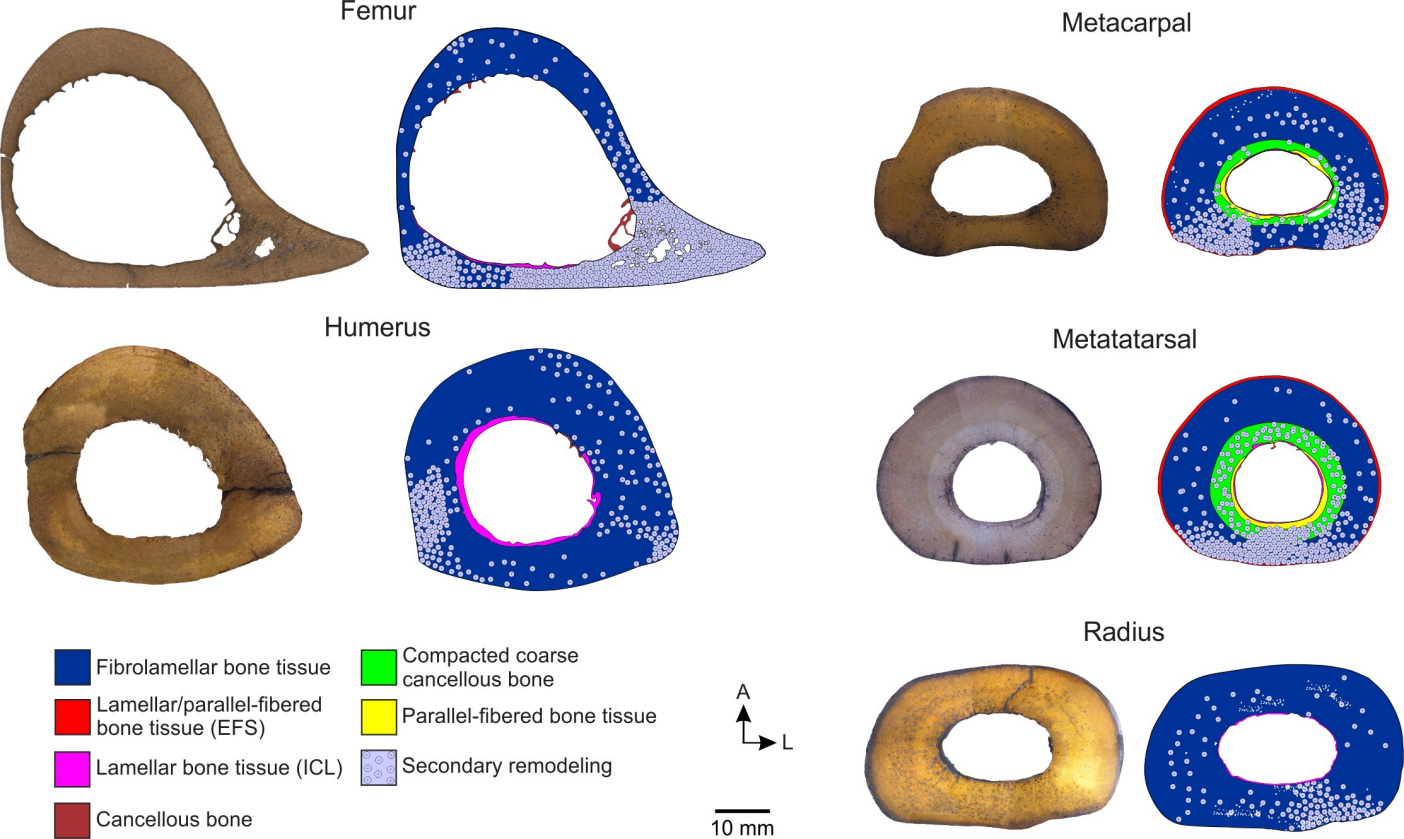
Chips and schematic cross-sections of *Equus occidentalis* skeletal elements showing the tissues variability. For each element analyzed, the image of the chip is shown (left) and a diagram showing the variability of the bone tissues observed (right). Note that EFS is not to scale. Abbreviations: EFS. external fundamental system. ICL. inner circumferential lamellae. A. anterior region. P. posterior region.

Transverse thin sections of all elements show a compact cortex surrounding a medullary region (Fig 1). The medullary cavity of all the specimens is empty; however, cancellous bone associated with some sectors of the perimedullary region of Mt-III, humeri, and femur is observed. Cancellous bone is mostly broken in humeri and Mt-III. Conversely, bony trabeculae in the femora are mostly intact. The trabeculae are constituted by several generations of secondarily deposited lamellar bone tissue and remains of secondary osteons. In all elements, an internal circumferential layer (ICL) of lamellar bone is recognized almost completely surrounding the medullary cavity (except in those areas in which the perimedullary tissue is formed by cancellous bone). The compact cortex exhibits a stratified pattern, in which two (i.e., humeri, femur, and radius) or four (i.e., Mc-III and Mt-III) successive layers separated from each other by reversal lines can be distinguished (Fig 1). Remodeling of primary bone tissue is evidenced by the presence of secondary osteons and resorption cavities (Fig 1).

#### Femur

**Fig 2:**
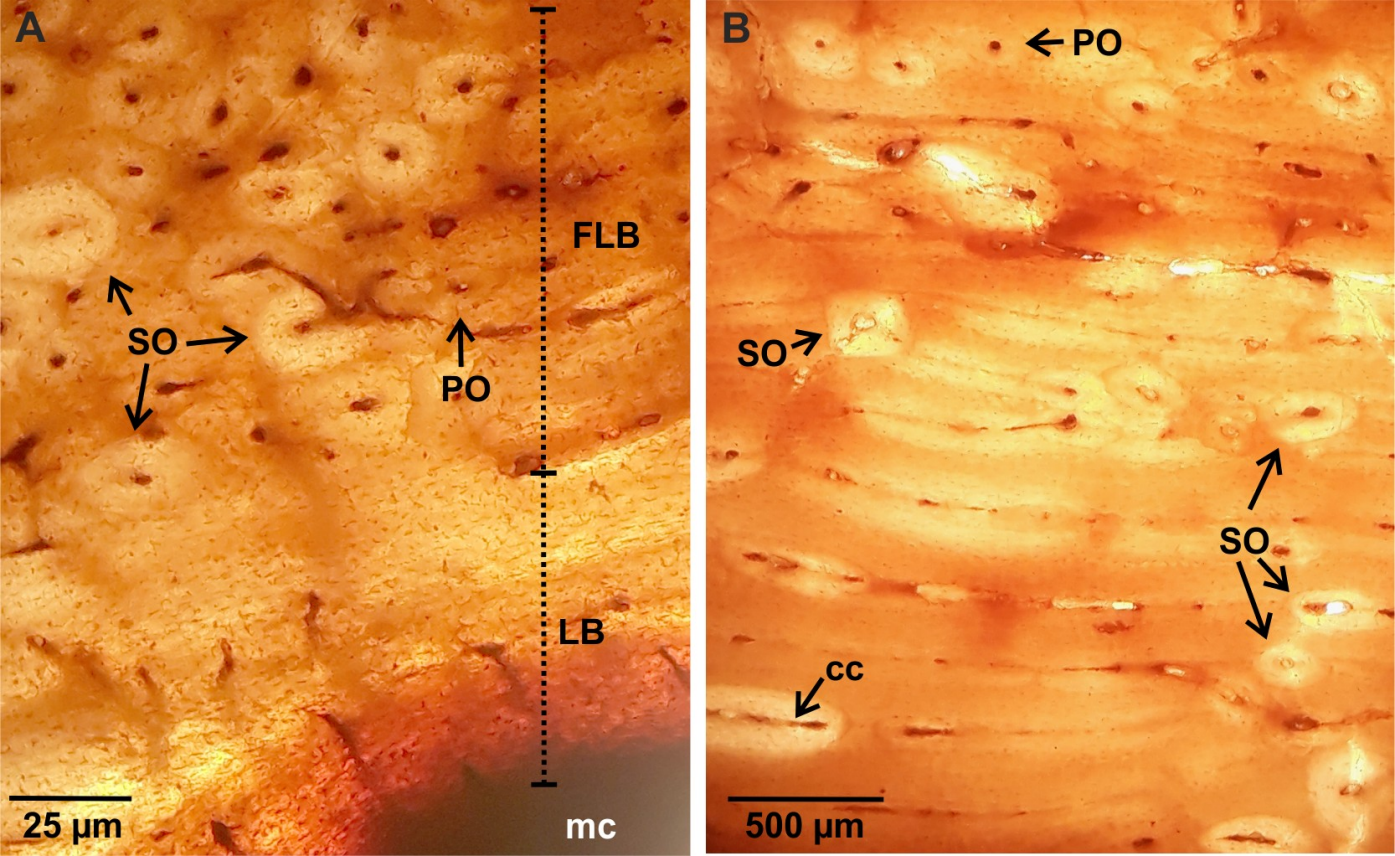
Femoral bone histology. (A). LACMHC 27421. ICL showing lamellar bone. Note the high remodeling of the fibrolamellar bone with some secondary osteons. (B). Detail of fibrolamellar bone showing primary osteons in circular rows, circumferentially oriented canals, and some secondary osteons. Abbreviations: cc. circumferentially oriented canals. FLB. fibrolamellar bone. LB. lamellar bone. mc. medullary cavity. PO. primary osteons. SO. secondary osteons. Images obtained under normal polarized light.

It shows a roughly triangular shape in cross-section. The ICL is thin (~0.05–0.70 mm) and surrounds the medullary cavity in the anterior and posterior regions (Fig 2A). It is traversed by radially oriented canals. Some secondary osteons are scattered in this layer. The primary bone is formed by fibrolamellar bone tissue, with primary osteons arranged into successive circumferential rows limited by thin concentric layers of woven-fibred bone tissue. Canals of the primary osteons anastomose with some circumferentially oriented canals and some radial canals, resulting in a laminar/plexiform vascular pattern (Fig 2B). The postero-lateral region is completely remodeled, showing dense Haversian bone with several generations of secondary osteons (Figs 1 and 2A). The density of secondary osteons decreases progressively from this region towards medial and anterior regions, where only isolated secondary osteons are observed (Figs 1 and 2B). Also, resorption cavities are observed in the perimedullary (postero-lateral) region, which are partially filled with lamellar tissue. No growth marks are identified in the compact cortex (Table 2).

#### Humerus

**Fig 3:**
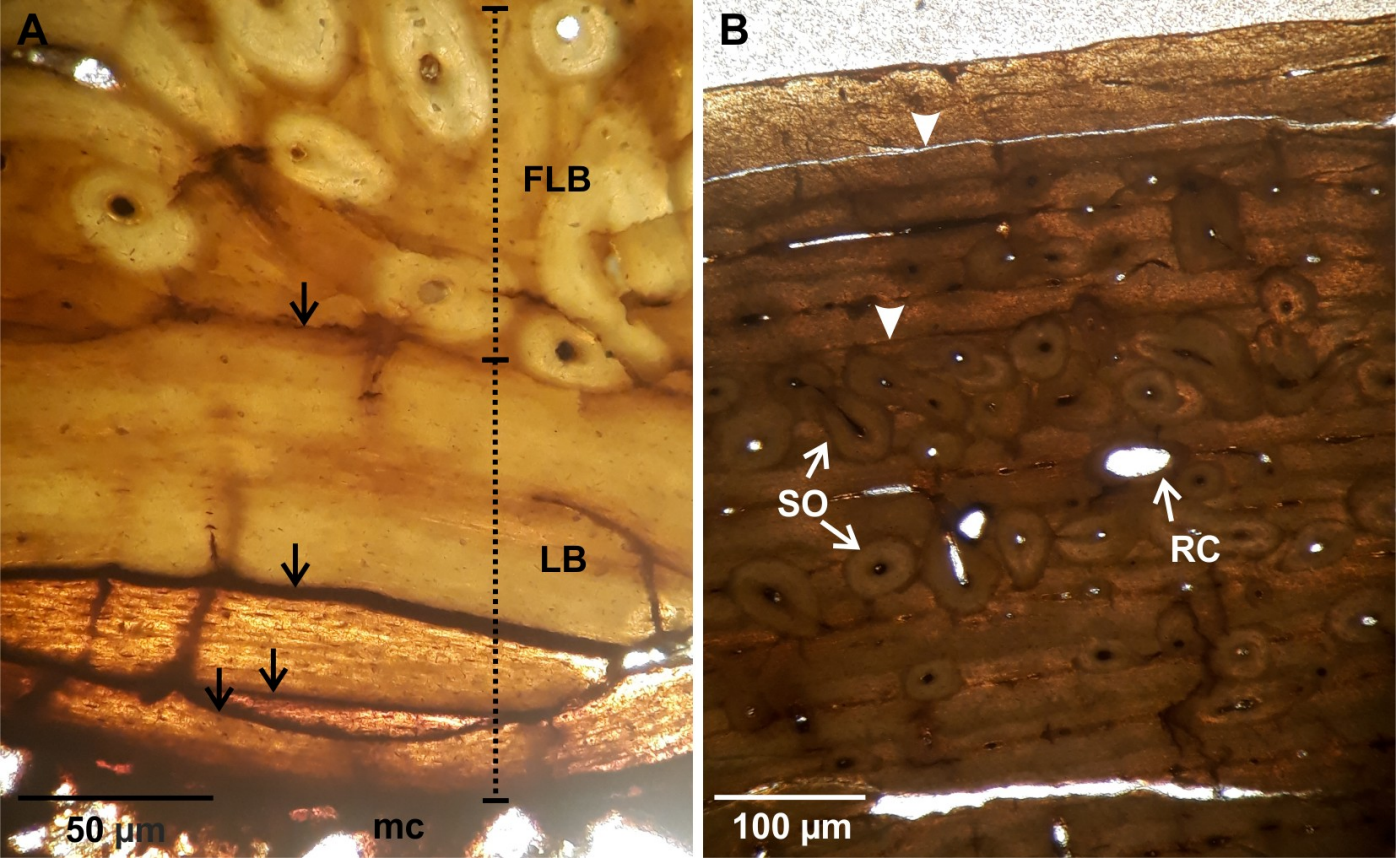
Humeral bone histology. (A). LACMHC 25297. ICL showing different generations of lamellar bone separated from each other by resorption line (black arrows). The image also shows a specific area of the fibrolamellar bone with remodeling, which is represented by multiple secondary osteons. Image obtained under cross-polarized light. (B). LACMHC 25346. Detail of the fibrolamellar bone showing remodeling represented by several generations of secondary osteons and resorption cavities. Two LAGs (black triangle) are identified in this cortex. Image obtained under normal polarized light. Abbreviations: FLB. fibrolamellar bone. LB. lamellar bone. mc. medullary cavity. RC. resorption cavities. SO. secondary osteons.

It shows a roughly circular shape in cross-section. A thin ICL of variable thickness (~0.20–2.05 mm) is observed in both specimens. This layer is formed by lamellar bone tissue deposited during different generations, as clearly indicated by the presence of resorption lines (Fig 3A). The external cortex is constituted by fibrolamellar bone tissue, with primary osteons arranged into successive circumferential rows limited by thin concentric layers of woven-fibred bone tissue. It is observed that the longitudinal canals of the primary osteons anastomose with circumferentially oriented canals and some radials canals (Fig 3A and 3B). Remodeling affects the entire compact cortex with different intensity according to the region, which is evidenced by the presence of secondary osteons and resorption cavities partially filled with lamellar tissue. In the medial and lateral regions, the bone is almost completely remodeled (Fig 1). In the lateral region, particularly in the outermost portion of the compact cortex, a high density of secondary osteons is registered. In the medial region secondary osteons are present in the entire cortex. Also, there are large resorption cavities in the lateral region, filled with lamellar tissue, which connect with the medullary cavity. The density of secondary osteons is lower in the anterior and posterior regions (Figs 1 and 3B). Sharpey’s fibers bundles oriented oblique to the bone surface are recorded in the outer portion of this cortex. LAGs are present in the external layer of both humeri (Table 2 and Fig 3B).

#### Radius

**Fig 4:**
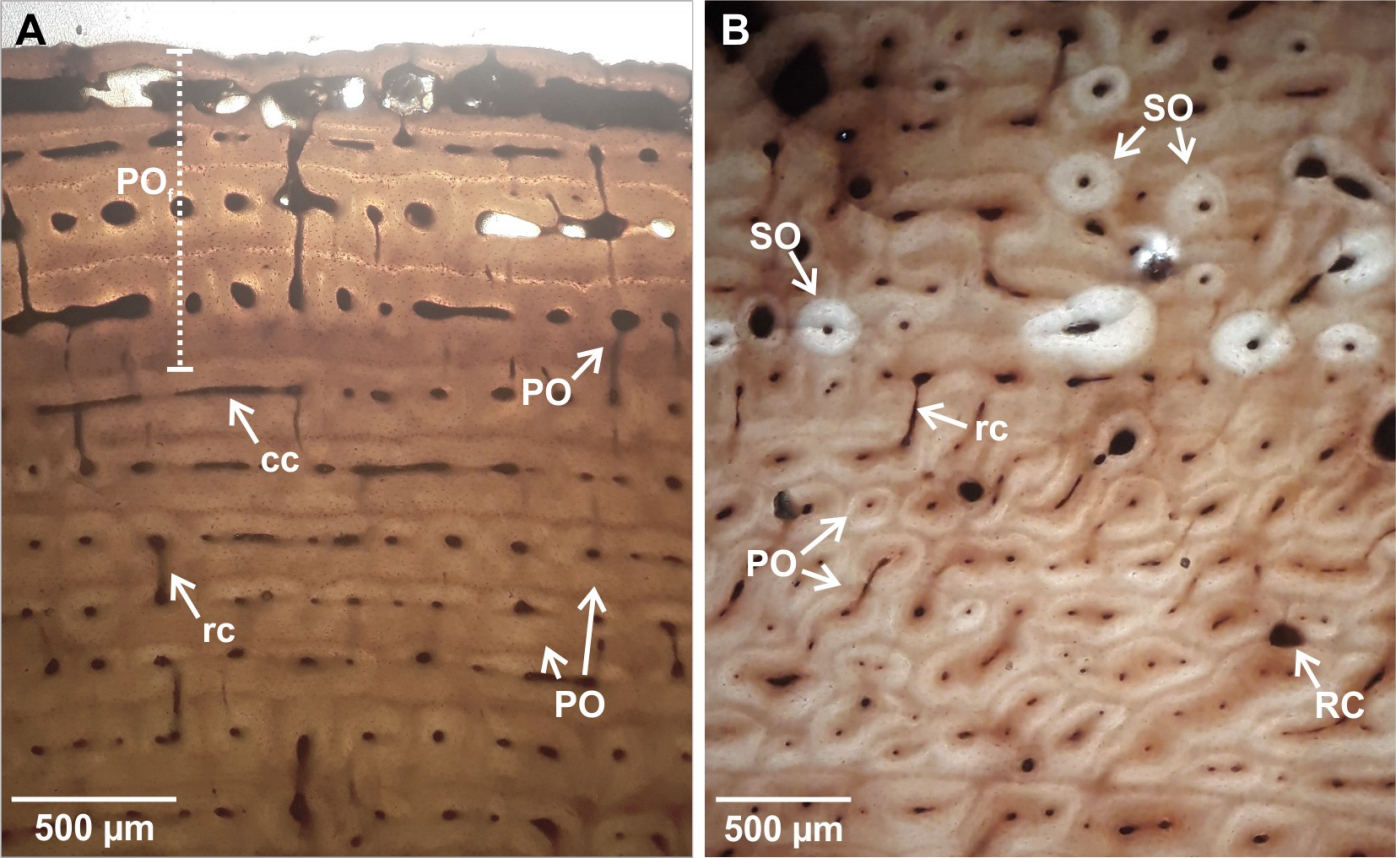
Radial bone histology. (A). LACMHC 6154. Fibrolamellar bone showing an inner portion represented by primary osteons anastomose with circumferentially oriented canals and some radial canals (plexiform pattern), and an external portion reflecting an incipient formation of primary osteons. (B). Detail of the inner portion showing the fibrolamellar bone with evidence of remodeling represented by some isolated secondary osteons and resorption cavities. Abbreviations: cc. circumferentially oriented canals. PO. primary osteons. POf. primary osteons in formation. RC. resorption cavities. rc. radial canals. SO. secondary osteons. Images obtained under normal polarized light.

It shows an oval shape in cross-section. The ICL is thin (~0.05–0.2 mm), avascular, and surrounds the medullary cavity in the lateral and posterior regions. The cortex is formed by fibrolamellar bone tissue, with primary osteons arranged into circumferential rows limited by thin concentric layers; the first three of these concentric layers look like bright lines under normal light. The vascular pattern varies according to the region. In the innermost portion, it is observed that the longitudinal canals of the primary osteons anastomose with some circumferentially oriented canals and some radial canals, resulting in a plexiform vascular pattern (Fig 4A). The outermost portion displays longitudinally oriented simple canals, filled by some lamellae, reflecting the incipient formation of primary osteons (Fig 4A). The remodeling is mainly restricted to the innermost portion of the posterior region and the inner and middle portion of the antero-lateral region. Some isolated secondary osteons and resorption cavities are distributed in the ICL and the outermost portion of the compact cortex (Figs 1 and 4B). No growth marks are identified in the compact cortex (see Table 2).

#### Metatarsal and metacarpal

**Fig 5:**
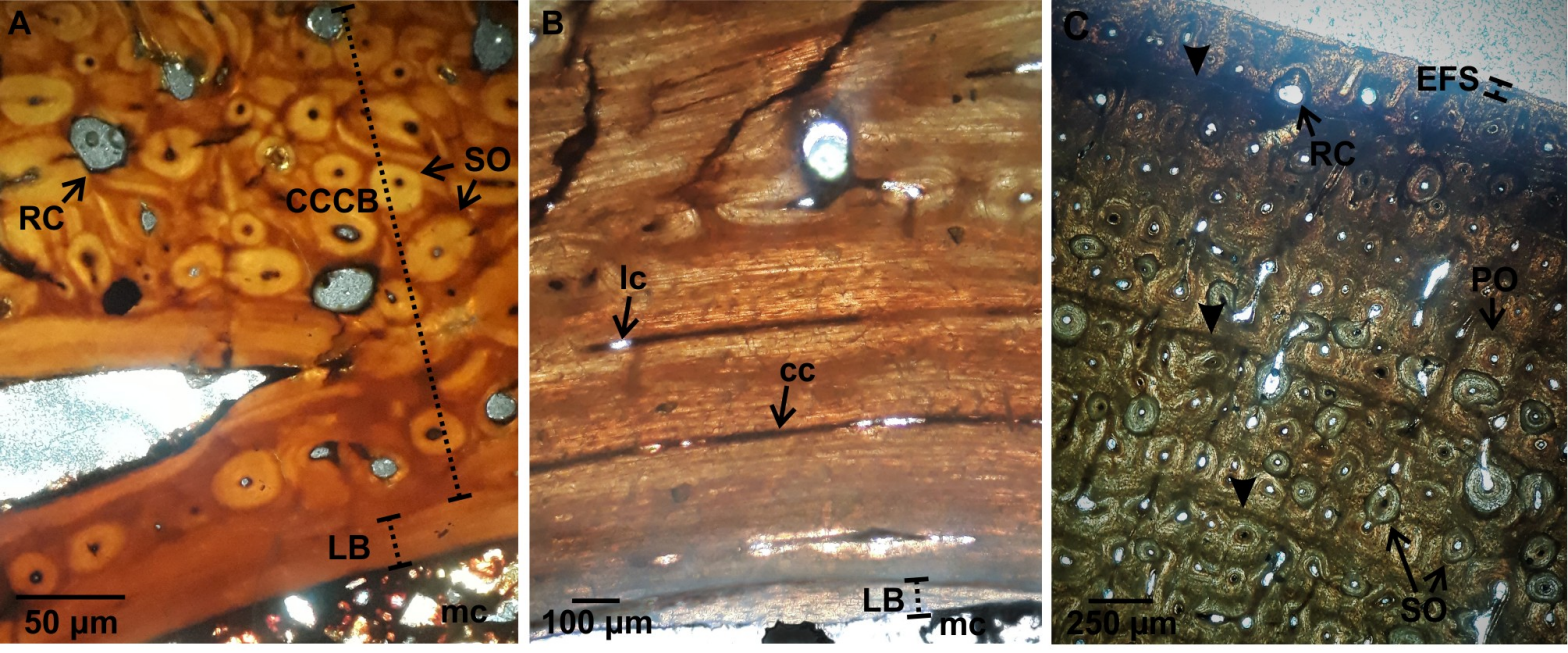
III Metacarpal bone histology. (A). LACMHC 26267. 381 ICL showing lamellar bone and CCCB highly remodeled. Image obtained under normal polarized light. (B). LACMHC 26263. Detail of the parallel fibered bone, showing longitudinal and circumferentially oriented canals. Image obtained under cross-polarized light. (C). LACMHC 26263. Fibrolamellar bone shows evidence of remodeling, represented by some secondary osteons and resorption cavities. Three LAGs (white triangle) are identifiable in this layer. Note that the most external layer is represented by parallel fibered bone (EFS). Image obtained under cross-polarized light. Abbreviations: CCCB. compact coarse cancellous bone. EFS. external fundamental system. FLB. fibrolamellar bone. LB. lamellar bone. mc. medullary cavity. PFB. parallel fibered bone. PO. primary osteons. RC. resorption cavities. SO. secondary osteons.

**Fig 6:**
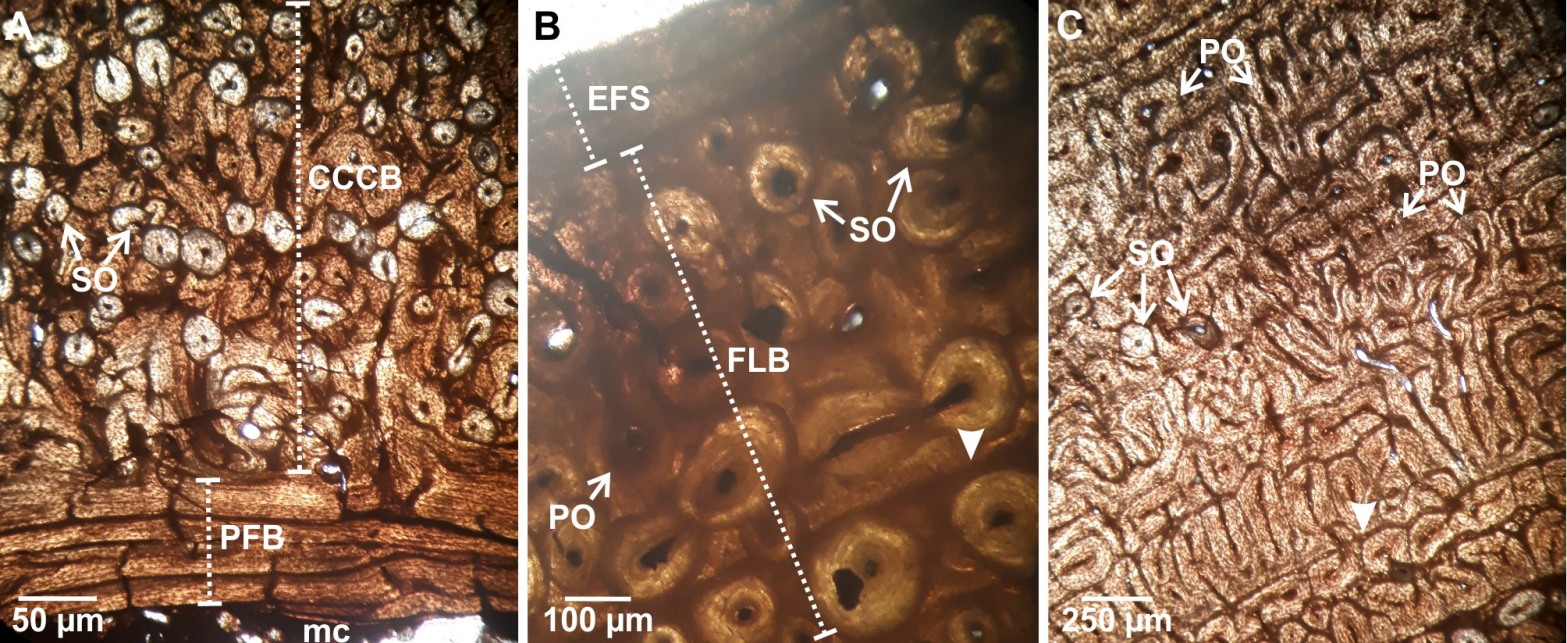
III Metatarsal bone histology. (A). Z 4697. The two observed layers include parallel fibered bone, with longitudinal and circumferentially oriented canals, and CCCB highly remodeled with several generations of secondary osteons. (B). Z 4697. Fibrolamellar bone remodeled, including some secondary osteons. One LAG (black triangle) is identifiable in this layer. Note that the most external layer is represented by parallel fibered bone (EFS). (C). Z 4697. Detail of the external cortex showing a CCCB layer following by fibrolamellar bone layer with evidence of remodeling, represented by some secondary osteons. One LAG (black triangle) is identifiable in this layer. Abbreviations: CCCB. compact coarse cancellous bone. EFS. external fundamental system. FLB. fibrolamellar bone. LB. lamellar bone. mc. medullary cavity. PFB. parallel fibered bone. PO. primary osteons. SO. secondary osteons. Images obtained under normal polarized light.

The shape in cross-section is oval in Mc-III, flattened in the posterior region, and roughly circular in Mt-III. The cortex of these elements is formed by four distinct layers, being the ICL the innermost of them (Fig 5A). The ICL has an irregular thickness (~0.04–0.45 mm in Mt-III; ~0.07–0.62 in Mc-III) in all specimens. The ICL is followed by a prominent layer constituted by parallel fibered bone tissue, with longitudinal canals in the anterior and posterior regions and with longitudinal and circumferentially oriented canals in the lateral and medial regions (Figs 5B and 6A). Some secondary osteons are observed in this layer. The following layer is formed by compacted coarse cancellous bone, in which large cavities filled with lamellar tissue give a convoluted aspect to the bone (Figs 5A and 6A). This layer is highly remodeled, showing several generations of secondary osteons and resorption cavities, which partially obscured the convoluted aspect. The next layer is formed by fibrolamellar bone tissue with primary osteons arranged into successive circumferential rows limited by thin concentric layers of woven-fibred bone. It is observed that the longitudinal canals of the primary osteons anastomose with some circumferentially oriented canals and some radial canals (Figs 5C, 6B and 6C). Remodeling in this layer, represented by isolated secondary osteons, is mainly observed from the inner to the outer portion of the posterior region. A variable number of LAGs is observed in the external layer of both elements (see Table 2 and Figs 5C, 6B and 6C). Also, in both Mt-III and one Mc-III an external layer constituted by avascular lamellar/parallel fibered bone tissue it is possible to observe, which corresponds to the external fundamental system (EFS) (Figs 5C and 6B). In both Mt-III, the EFS includes Sharpey’s fibers bundles oriented obliquely to the bone surface.

### Histotaphonomy

The macroscopic examination (Table 3, S2 Fig) revealed that most of the studied fossil bones do not have cracking or flaking on their surfaces, which is consistent with weathering stage 0, except for Z 4697 and LACMHC 25297 that show stage 1. No abrasion evidence (stage 0) is present in the sampled specimens. Fossils exhibit both fresh biostratinomic and fossil-diagenetic fractures. All specimens are characterized by presenting the typical black color of the vertebrate remains from this site, which is related to the impregnation with asphalt.

**Table 3:** Macro- and microtaphonomic features of the different *Equus occidentalis* specimens.

		LACMHC 25346 (humerus)	LACMHC 25297 (humerus)	LACMHC 6154 (radius)	LACMHC 27421 (femur)	LACMHC 26263 (Mc-III)	LACMHC 26267 (Mc-III)	Z 4697 (Mt-III)	Z 4657 (Mt-III)
Macrotaphonomic features	Weathering	Stage 0	Stage 1	Stage 0	Stage 0	Stage 0	Stage 0	Stage 1	Stage 0
	Abrasion	Intact	Intact	Intact	Intact	Intact	Intact	Intact	Intact
	Type of fracture	Fossil-diagenetic	Biostratinomic	Fossil-diagenetic	Fossil-diagenetic	Biostratinomic	Fossil-diagenetic	Biostratinomic	Fossil-diagenetic
Microtaphonomic features	OHI	5	5	5	5	5	5	5	5
	Enlarged canaliculi	-	-	X	-	-	-	X	X
	Microtunneling	-	-	X	-	X	-	-	X
	Central radial microcracks	X	-	X	-	X	-	X	-
	Circumferential microcracks	X	-	X	-	X	-	X	-
	Peripheral radial microcracks	-	-	-	-	-	-	-	-
	Radial microcracks	-	X	-	X	-	-	-	X
	Circumferential fissures	ICL	ICL and LAGs	-	-	LAGs	ICL	ICL and LAGs	-
	Fissures	X	X	X	X	X	X	X	X
	Medullary cavity infilling	X	X	X	X	X	X	X	X
	Microstructural cavities infilling	X	X	X	X	X	X	X	X

Histotaphonomic features (Table 3) include alterations observed under light microscope, petrographic microscope, and scanning electron microscope. Specimens show a very well preservation of the bone microstructure, only affected by minor changes. The identified modifications comprise enlargement of canaliculi, microtunelling, radial and circumferential microcracks on secondary osteons, fissures, and microstructural cavities infilling. It is worth noting that none of the examined samples show evidence of bacterial attack altering the original characteristics of the bone tissue. All samples can be assigned to the stage 5 of the OHI proposed by Hedges et al. [29]. The analysis reflects some taphonomic differences among the specimens of the different pits.

**Fig 7:**
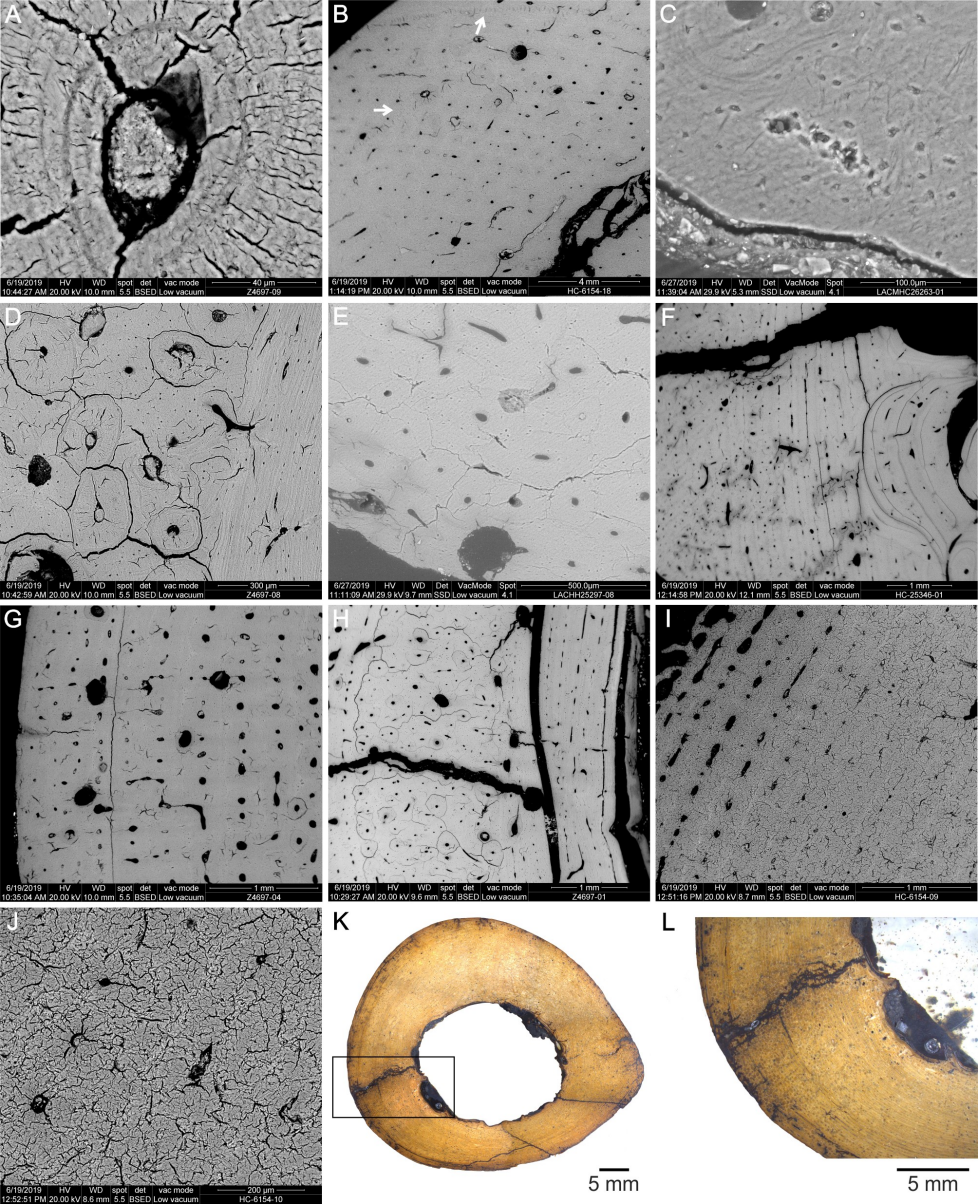
Histotaphonomic features of *Equus occidentalis* skeletal elements. A. Mt-III Z 4697, primary osteons of the external layer showing enlarged canaliculi. B. Radius LACMHC 6154, zig zag microtunnels in the external layer, affecting primary and secondary osteons. C. Mc-III LACMHC 26263, branched and unbranched straight microtunnels distributed in the external layer. Note the development of a slightly crumbly texture. D. Mt-III Z 4697, secondary osteons showing central radial microcracks associated to circumferential microcracks located in the peripheral portion. E. Humerus LACMCH 25297, secondary osteons showing radial microcracks located in their borders, forming bridges with the adjacent osteons. F. Humerus LACMCH 25346, circumferential fissures following resorption lines. G. Mt-III Z 4697, circumferential fissures following LAGs. H. Mt-III Z 4697, fissures unrelated with the bone histology, cutting the compact cortex and filled with asphalt-impregnated clastic material. I. Radius LACMHC 6154, development of “craquelure texture” associated to areas with development of enlarged canaliculi and central radial and circumferential microcracks. J. Detail of the affected area. K. Humerus LACMHC 25297, cross-section of the sample showing the filling of different types of cavities with asphalt-impregnates clastic material. L. Detail of the filling in medullary and microstructural cavities and fissures.

Enlarged canaliculi are present in some primary osteons of the external layer in LACMHC 6154, while in Z 4697 (Fig 7A) and Z 4657 they affect some secondary osteons of the two most external layers. In all cases, these canaliculi are apparently empty.

Microtunelling affects three specimens. Zig-zag microtunnels, with different orientations, limited to a small sector of the external layer and affecting primary and secondary osteons are present in LACMHC 6154 (Fig 7B). In LACMHC 26263 there are branched and unbranched straight microtunnels, mainly parallel to each other, which appear limited to a small sector of the external layer (Fig 7C). All of them are empty, with unaltered borders, and apparently do not affect nearby osteons. The affected area shows a slightly crumbly texture (Fig 7C). In Z 4657, unbranched straight microtunnels, mainly parallel to each other and limited to a small sector of the external layer, are observed. All of them are empty, with unaltered borders, and apparently do not affect nearby osteons.

Microcracking related to the bone histology affects most of the specimens, except LACMHC 26267. In LACMHC 25346, LACMHC 6154, and Z 4697, central radial microcracks are present in secondary osteons, radiating from the walls of the Haversian canals to the peripheral zone of the osteon (Fig 7D). In LACMHC 6154 and Z 4697, these microcracks are linked to circumferential microcracks located in the peripheral portion, following the mineralized cement line, which generate the separation between adjacent osteons (Fig 7D). Peripheral microcracks spreading inwards from the borders of a secondary osteon are absent in these specimens. Radial microcracks located in the borders of secondary osteons, forming a bridge with the adjacent ones, are present in LACMHC 25297, LACMHC 27421, LACMHC 26263, and Z 4657 (Fig 7E). In most specimens, different types of microcracks are empty, except in LACMHC 25297, in which they seem to be partially filled with different mineral components.

In the specimens LACMHC 25346, LACMHC 26267, LACMHC 26263, LACMHC 25297, and Z 4697, there are circumferential fissures larger than the microcracks described above, also linked to the bone histology. They are following resorption lines of the ICL (Fig 7F) and LAGs of the external layer (Fig 7G). These types of fissures are partially filled with asphalt-impregnated clastic material and different mineral components.

Other fissures unrelated with the bone histology are present in all specimens, partially or completely cutting the compact cortex and without any preferential direction. Most fissures are filled with asphalt-impregnated clastic material (Fig 7H). Several of them cut secondary osteons with microcracks. In LACMHC 25346 and LACMHC 25297, some of them might be internal prolongations of the weathering splitting.

A “craquelure texture” of the bone tissue is present in different sectors of the sample, both in LACMHC 6154 and Z 4697 (Fig 7I and 7J). This feature seems to be associated to the areas with more development of enlarged canaliculi and central radial and circumferential microcracks.

A feature shared by all the specimens is the presence of asphalt-impregnated material infilling, partially or completely, the medullary and microstructural cavities (Fig 7K and 7L). Clastic material and different mineral components (mainly carbonates, quartz, feldespars, and opaque minerals) constitute the filling. In most cases, microstructural cavities are completely filled.

## Discussion

### Life history traits, physiology, and growth pattern

We studied eight samples corresponding to limb bones (i.e., humerus, radius, femur, Mt-III, and Mc-III), which were recovered from several pits (and bearing-levels with variable chronology; see above, Table 1) and assigned to different individuals (Tables 1 and 2). Considering that all materials used were incomplete, the sector where the thin sections were made varied according to the preserved portion of each specimen. Finally, some thin sections were thicker than standard paleohistological sections due to preparation difficulties by the presence of asphalt in both the outer surface and microstructural cavities. However, beyond these situations, a first detailed approach of the *E*. *occidentalis* osteohistology could be carried out in this work.

Observations performed in this study reflected that all skeletal elements are fundamentally characterized by the presence of fibrolamellar bone tissue, although with differences in the vascular pattern, coinciding, in general terms, with the descriptions of other equids, both extant [18, 25, 55–60] and extinct [18, 26–28, 57, 61, 62]. The overall presence of this type of tissue suggests that this species has, during early stages of the growth, a relatively fast rate of bone deposition. Cuijpers and Lauwerier [63] indicated that both horses and cattle grow relatively fast (in comparison with humans) due they have to be fully grown at 3–4 years. Variations recorded among specimens could be related to specific growth rate, size, growth dynamics, or the biomechanical factors, according to particular characteristics of each skeletal element and each individual (see [58, 59, 64]).

Mt-III (Z 4697 and Z 4657) and Mc-III (LACMHC 26263 and LACMHC 26267) have a layer of compacted coarse cancellous bone. This secondary tissue is linked to the modeling (i.e., remodeling *sensu* Enlow [65]) processes generated by the longitudinal growth of the skeletal element and by compaction and drift of the medullary cavity during ontogeny [21, 66]. Its presence in all analyzed metapodials would be linked to the fact that, according to the portion preserved in each specimen, the cuts could not be made exactly in the mid-diaphysis. This finding provides new information on this type of tissue, since it was recently reported for the first time in extinct equids, specifically in *Hipparion* specimens [27].

The EFS was registered only in two Mt-III (Z 4697 and Z 4657) and one Mc-III (LACMHC 26263) (Table 2). In all cases it appears after the deposition of the last LAG of the fibrolamellar layer (see below, Skeletochronology). This cortex evidences the end/decrease of periosteal bone growth [27], although the interpretation of its origin is controversial. Some authors consider that its presence in mammals reflects the attainment of sexual maturity (e.g., [20, 58, 62, 67, 68]), while others propose a relationship with the attainment of skeletal/somatic maturity (e.g., [26, 46, 61, 69–71]). Particularly for equids, it was mentioned that the timing of deposition of this tissue in the femur is causally correlate with the age of the first reproduction [58, 68], while the meaning in other skeletal elements is still poorly know ([62]; but see [26]). In our case, no interpretations are made of the absence/presence of this tissue until a larger number of samples of different skeletal elements and a diversity of ontogenetic stages are available.

Secondary remodeling signs (i.e., secondary osteons and resorption cavities) were identified in all specimens, although with differences in the location of the affected areas and the intensity, which would suggest that the development of this process is particular for each skeletal element (see [26]). In this sense, it was observed that a same skeletal element (i.e., humerus, Mt-III, Mc-III) showed similar features in different specimens. In all samples, it was noted that the relative density of secondary osteons varies according to the location, registering areas with higher concentrations (even with the development of Haversian tissue) and areas where they were scarce and scattered. The remodeling pattern observed in each type of skeletal element is, in general lines, similar to that described in other works for limb bones of both extant (e.g., [18, 25, 56–59]) and extinct (e.g., [18, 26, 27, 55, 57, 61]) equids.

This remodeling process would be associated to changes occurred during ontogeny (i.e., remodeling intensity increase with age) and to biomechanical factors (see [26, 58]). The variations observed in the same sample would indicate that some areas are subjected to compression loads (increase of secondary osteon density) while in others the tension strains are predominant (decrease of secondary osteon density) [26, 56, 57, 59].

### Skeletochronology

Skeletochronological studies, based on the presence of cyclical growth marks, help to understand different life history traits of the species, such as longevity, age at maturity, and growth strategy, among others (e.g., [46, 59, 67]). Cyclical growth marks identified in the skeletal elements of *E*. *occidentalis* correspond to LAGs, which represent cyclical growth marks that evidence moments of cessation of growth [19, 21]. Taking into account that LAGs register annual cycles of growth (e.g., [24, 44] and references therein), we estimate here the minimum age of the individuals at the death time based on the number of marks identified in each sample. It should be noted that, in some cases, secondary remodeling can obscures the presence of growth marks and, therefore, the counting may be underestimated [46].

Studies performed in extant and extinct equids suggest that femora and metapodials are valuable skeletal elements for skeletochronological analysis (see [25, 26, 58, 59, 62]). We identified LAGs in six specimens (from a total of eight), including two humeri, two Mc-III, and two Mt-III (Table 2). The higher number of LAGs identified was 3, in both Mc-III.

No LAGs were observed in the radius (LACMHC 6154) and femur (LACMHC 27421). The absence in the first one could be related to the ontogeny, since the histological features (i.e., incipient formation of primary osteons, presence of vascular canals open towards the sub-periosteal margin, sub-periosteal margin with irregular contour, low remodeling) reflect a juvenile stage. The second one has histological features (i.e., absence of vascular canals opens towards the sub-periosteal margin, sub-periosteal margin with smooth contour, high remodeling) corresponding to a more advanced ontogenetic stage (subadult/adult), so the absence would be the result of the remodeling process.

The number of identified LAGs varied according to the skeletal element, registering was one (LACMHC 25346) and two (LACMHC 25297) in the humeri, two in the Mt-III (Z 4697 and Z 4657), and three in the Mc-III (LACMHC 226263 and LACMHC 26267). Interestingly, both Mt-III and one Mc-III developed an EFS located after the deposition of the last (second and third respectively) LAG; this record agrees with the descriptions made for metapodials of other extinct equids (see [26, 61]). However, as it was mentioned, we do not discuss here the significance of this tissue.

Taking into account the characteristics of the sample, the growth curve was not reconstructed. The skeletal elements with more than two LAGs are represented by only two specimens (Mc-III LACMHC 226263 and LACMHC 26267), which could not be sampled in the same portion and, furthermore, one of them was partially incomplete. This situation makes it difficult to interpret the results obtained.

### Taphonomic history reconstruction

Several studies proposed an entrapment scenario to explain the origin of the mammal assemblages recovered in the different pits of the Rancho La Brea site, which involve both herbivores and carnivores that exploited their carcasses (e.g., [10]). However, other possible taphonomic histories should not be ruled out, since it is still necessary to deepen in the evaluation of diverse processes and agents that may have affected the bone remains both before and after burial. In this work, we provide new taphonomic information from a novel perspective (histotaphonomy), which is based on the analysis of the modifications that affected the microstructure of the *E*. *occidentalis* bone remains.

From a macroscopic viewpoint, the taphonomic features identified in the remains here studied (Table 3) coincide with information obtained in previous works using material from different pits (see [2, 5, 14] and references therein). All specimens are completely impregnated with asphalt, which results in a dark coloration; different authors (e.g., [72–74]), proposed that the asphalt favored the excellent preservation of the materials from this site, practically in its original state. Null and slight weathering degrees allow estimating a relatively short permanence time in the surface of the specimens exposed to different atmospheric agents; according to Spencer et al. [5], carcasses of the animal entrapped in the tars were, in a first moment, only partially submerged. Based on entomological information, Holden et al. [9] suggested that carcasses could remain exposed in the surface of the pits for at least 17–20 weeks before being fully submerged in the asphalt; however, we do not record insect trace damage in the specimens analyzed. Null abrasion degree in all specimens reflects a short time of interaction between bones and sedimentary particles or very low intensity of the process. Despite the possible development of water transport events of short duration in the area (i.e., flash floods; [75]), Spencer et al. [5] mentioned that fluvial action was not significant in the formation of the assemblage from the Pit 91 (but see [14]). The types of identified fractures indicate that the specimens were affected by destructive processes in different stages of the taphonomic history, both before and after burial.

From a microscopic viewpoint, diverse biotic and abiotic agents and processes may modify and/or destroy the original histological features of bone remains during different stages of the taphonomic history. These changes vary according to the particular characteristics of the preservational contexts ([30–32, 54, 76] and references therein). We identified here diverse modifications that slightly altered the microstructure of the bone remains. Differences recorded among samples would be linked to the taphonomic history of each specimen and the conditions of each pit.

Canaliculi enlargement was observed in three specimens of different pits, including a radius and two Mt-III (Tables 1 and 3). This process affected both primary and secondary osteons, in all cases located in the most external layers of the compact cortex. It has been linked with soil corrosion by highly humid acidic substrate and/or biogenic corrosion by moss, algae, and lichen (e.g., [30, 31, 77, 78]). Although there are no data on the dominant conditions in the pits here analyzed, Kim and Crowley [79] recorded slightly acidic conditions for the asphalt-permeated soil from Pit 91, which would support the interpretation of soil corrosion. On the other hand, from the biological viewpoint, there is no additional evidence (i.e., alteration of the outer surface of bone remains) in the bone remains considered in this study to propose that the modifications are related to the acid fluids from elements of the vegetation that penetrated into the bone; additionally, previous taphonomic works do not either mention macroscopic alterations that can be linked to these agents. It probably took place during the early diagenesis.

Microbial attack is one of the key dominant processes altering and destroying bone tissues during the early stages after the death of the animal. They are driven by several factors, including the death history of an animal, its decomposition trajectory, and the depositing environment itself [32, 80, 81]; however, it is a process that remains poorly understood for terrestrial environments [82]. Some authors (e.g., [83, 84]) suggested that the different microbial alterations of bone remains are, in all cases, the result of bacterial attack; however, we follow here the traditional proposal that relates different types of modification with different agents (e.g., [30, 51]). In the samples studied, bioerosion traces that can be related to microbial attack are scarce and little developed, which would have favored the excellent preservation (stage 5 of Hedges et al. [29]) of the bone microstructure.

Recorded traces include microtunnels located in the most external layer of the compact cortex (penetrating only a few microns). They were observed in three specimens of different pits, including a radius, a Mc-III, and a Mt-III (Tables 1 and 3). The general aspect (i.e., shape, size, crumbly texture, location) of these microtunnels relate them to “Wedl tunneling” (*sensu* [50]) and, therefore, suggest fungal attack as their probable origin (see [30, 31, 50, 85–89]). Particularly, microtunnels with a zig-zag trajectory are similar to the traces produced by the fungal genus *Mucor*, cultured on modern bones in laboratory (see [30, 31]). Colonization of bones by fungi, and the consequent microstructure modification, occurs under favorable environmental conditions (i.e., presence of oxygen and moisture) [51, 90, 91]; the alteration of the bones by fungi may occur very quickly after death, in a matter of a few days (i.e., *Mucor* fungus; [90]). In this case, there is no additional evidence to determine with certainty the moment (biostratinomic or fossildiagenetic stages) in which the alterations were originated. The absence of crumbly texture in most of the specimens (except the Mc-III LACMHC 26263) suggests that, in general, the intensity of the fungal attack was slight.

Studied specimens do not show evidence (e.g., hyper-mineralized zones -non-Wedl microscopic focal destruction- containing networks of small pores and thin canals; [30, 32, 77, 80, 92] and references therein) that can be related to the attack of both indigenous and exogenous bacteria. This point is particularly interesting since the bacterial attack is a very common process recorded in archeological and paleontological bone remains, which occurs quickly after death [51, 82]. Besides, it was indicated that, nowadays, bacteria inhabit the tar seeps of Rancho La Brea and they seem to have an active participation in the decay of tissues [79].

Kim and Crowley [79] analyzed two pits (91 and 101) of the Rancho La Brea site and observed simple microbial community structures, including diverse types of bacteria, with some differences possibly due to the particular conditions (i.e., Pit 91 asphalt-permeated soil: greater concentration of petroleum hydrocarbons, slightly acidic, and relatively low salinity and metal content; Pit 101 water suspensions of asphalt-permeated soil: alkaline (pH 8.4), and high concentration of salts and metals) of each pit. These authors suggested that this type of extreme environmental context (i.e., lack of air and water, presence of highly recalcitrant carbon sources, and high concentrations of potentially toxic metals and chemicals) is highly selective, which require specialized adaptations and, therefore, limits the microbial development.

Brown et al. [12] made an actualistic experiment in an undisturbed tar seep, using bobcat (*Lynx rufus*) carcasses, to determine the progress of microbial faunal changes and tissue decay in this anaerobic environment. These authors demonstrated that carcasses submerged in the asphalt are rapidly skeletonized (i.e., loss of all muscle tissue, tendons, and ligaments, leaving only a viscous mat of hair and skin collagen remaining around the bones), after 2–3 months, due to the activity different microbial communities, including bacteria. Differences in the composition of the communities among pits and animal decay were identified, suggesting specializations on utilizing available resources (see also [93]). The succession patterns of the identified microbial communities would indicate that the microbes most involved in the skeletonization of the carcasses are originated from the liquid surface tar.

In this context, it is worth asking: why do we not observe evidence of bacterial attack of the bone tissue at microstructural level? The consideration of different factors, acting independently or in combination, can help to understand and explain this situation: 1) the extreme conditions of this type of environment limit the growth and diversity of bacteria (see [79]), and may favor the development of specialized taxa (see [12]), in this case with a participation possibly limited to the decay of soft tissues; 2) dismemberment or skeletonization shortly after death, particularly due to the activity of predators and/or scavengers (including both carnivore mammals and insects; see [5, 9]), prevents or reduces the putrefaction of the bones and, therefore, the bacterial attack; and 3) a quickly impregnation of the outer surface and infilling of the microstructural cavities with asphalt would limit the invasion of bacteria inside the bone through the vascular network. At the moment, however, it is not possible to provide more information on this phenomenon, since there is little available information on the biology of the bacteria communities that live in tar seeps and their participation in the tissue decay as well as on the conditions of the tar seep during the late Pleistocene and the accumulation history of the bones.

The good preservation of bones due to the absence of bacterial attack may result in an increased source of nutrients for the saprophytic fungi of the soil (i.e., *Mucor*, see [30, 31, 51, 77, 90, 91]). Although some of the analyzed specimens have traces assignable to fungal activity, the available evidence does not allow confirming if fungi used the bones for feeding or merely as a substrate.

Microcracking affecting bone histology provides information on the accumulation and burial environment (e.g., [52–54, 94–97]). The sample studied includes specimens from different pits (Tables 1 and 3) with two patterns of microcracking that affect the secondary osteons. In both cases, microcracks were produced during the early diagenesis.

Radial microcracks that appear on the outer borders of the osteons and connect them with adjacent osteons are originated in skeletal elements fossilized under water [52, 53, 94]. Central radial microcracks that extend outwards from the walls of the Haversian canals indicate desiccation of the skeletal elements that were fossilized under dry, terrestrial conditions [54]. Associated with these last microcracks, we identified in all the specimens circumferential microcracks located in the peripheral zone and following the mineralized cement line, which indicate buildup of shrinkage stress due the water loss continuity; the absence of peripheral radial microcracks associated with the circumferential ones suggests that the desiccation intervals were relatively shorts and, therefore, the shrinkage did not continue (see [54]). The variations recorded in the type and intensity of microcracking reflect clear differences in the preservation conditions among pits.

Some specimens show circumferential fissures following debility zones of the bone microstructure, such as resorption line and LAGs (see also [26]), both in the ICL and the external layer (Table 3). These fissures were probably also formed during the early diagenesis. Finally, there are other fissures that are independent of bone histology (Table 3); some of them are probably related to weathering and originated during the pre-burial stage, while others affect secondary osteons with microcracks and were formed after the early diagenesis.

Asphalt-impregnated clastic material fills, in all specimens, the medullary and microstructural cavities and the fissures that cut the compact cortex (Table 3). According to the evidence, the filling process appears to have affected bone remains in different stages (pre- and post-burial) of the taphonomic history. It is possible to propose that the permineralization with this asphalt-impregnated clastic material could explain the good preservation of the bone microstructure of the specimens from this site.

## Conclusions

We present here the first comprehensive osteohistological and histotaphonomic study performed in bone remains from different pits of the emblematic Quaternary fossiliferous locality of Rancho La Brea. It provides, from a different perspective, preliminary novel information to know different life history aspects of *Equus occidentalis*, one of the most abundant taxa in this site, and to better understand the origin and taphonomic histories of the vertebrate assemblages preserved in this particular terrestrial preservational context.

From a paleobiological viewpoint, we characterize and interpret the bone microstructure of different skeletal elements. Even when all the specimens of the studied sample were broken, this osteohistological analysis provides relevant information on *E*. *occidentalis* and allows to make detailed comparison with other extant and extinct equids. The overall presence of fibrolamellar tissue reflects a relatively fast rate of bone deposition during the early stages of the growth. The EFS identified shows end/decrease of periosteal bone growth in different moments of the ontogeny according to the skeletal element. In general lines, the identified features coincide with those mentioned for other extant and extinct equids. Secondary remodeling is present in all skeletal elements, but with particular differences in intensity and location. Cyclical growth marks (represented by LAGs in this case) allowed us to propose preliminary skeletochronological interpretations, mainly related to the minimum age at the moment of death of the studied specimens.

From a taphonomic viewpoint, we describe diverse agents and processes not previously mentioned for the remains of this site, which affected the original bone microstructure during pre- and post-burial stages. It is important to highlight that this analysis includes some pits not considered in previous taphonomic studies. The obtained results reflect differences among pits, suggesting variations in the environmental conditions and taphonomic histories. Differences are even recorded among specimens from a same pit; however, the size of the studied sample makes it difficult to raise interpretations respect to these variations. All the specimens show slight modifications, which favored the excellent preservation of the bone microstructure; this aspect is concordant with the macroscopic features of the remains and endorses the consideration of Rancho La Brea as a *lagerstätte*.

Considering the problems that arose during the elaboration of the thin sections due to the asphalt present in the bone remains, filling both cavities and fissures, we had to propose modifications to the traditional techniques. This new methodological variant is optimal to work with vertebrate fossils preserved in tar seeps, since it allows making complete thin sections without altering the original features of the bone microstructure.

## Supporting information

S1 Fig Schematic diagram of the different skeletal elements studied showing the position in which the thin section was made. (TIF)

S2 Fig Main macrotaphonomic features observed in the specimens of the studied sample. (A). Humerus LACMHC 25346, showing stage 0 of weathering and fossil-diagenetic fracture. (B). Mc-III LACMHC 26263, showing stage 0 of weathering and biostratinomic fracture. (C). Mt-III Z4697, showing stage 1 of weathering and fossil-diagenetic fracture. (D). Humerus LACMHC 25297, showing stage 1 of weathering and fossil-diagenetic fracture. Note that all the specimens show black color related to the impregnation with asphalt. The specimens do not show abrasion evidence.
(TIF)

## References

[1] Marcus LF, Berger R. The significance of radiocarbon dates for Rancho La Brea, in: Martin S, Klein RG, editors. Quaternary extinctions. Tucson: University of Arizona Press; 1984. p. 159–188.

[2] Friscia AR, Van Valkenburgh B, Spencer L, Harris J. Chronology and spatial distribution of large mammal bones in Pit 91, Rancho La Brea. Palaios. 2008;23(1):35–42. doi: 10.2110/palo.2005.p05-143r

[3] O’Keefe FR, Fet EV, Harris JM. Compilation, calibration and synthesis of faunal and floral radiocarbon dates, Rancho La Brea, California. Contrib. Sci. 2009;518:1–16.

[4] Holden AR, Southon JR. Radiocarbon dating and stable isotopic analysis of insect Chitin from the Rancho La Brea Tar pits, southern California. Radiocarbon. 2016;58 (1):99–113. 10.1017/rdc.2015.9

[5] Spencer L, Van Valkenburgh B, Harris JM. Taphonomic analysis of large mammals recovered from the Pleistocene Rancho La Brea tar seeps. 2003;29(4):561–575. 10.1666/0094-8373(2003)029<0561:taolmr>2.0.co;2

[6] Coltrain JB, Harris JM, Cerling TE, Ehleringer JR, Dearing M-D, Ward J, et al. Rancho La Brea stable isotope biogeochemistry and its implications for the palaeoecology of late Pleistocene, coastal southern California. Palaeogeogr Palaeoclimatol Palaeoecol. 2004;205(3–4):199–219. 10.1016/j.palaeo.2003.12.008

[7] DeSantis LRG, Crites JM, Feranec RS, Fox-Dobbs K, Farrell AB, Harris JM, et al. Causes and consequences of Pleistocene megafaunal extinctions as revealed from Rancho La Brea mammals. Curr Biol. 2019;29(15):2488–2495.e2. doi: 10.1016/j.cub.2019.06.059 31386836

[8] Fuller BT, Southon JR, Fahrni SM, Farrell AB, Takeuchi GT, Nehlich O, et al. Pleistocene paleoecology and feeding behavior of terrestrial vertebrates recorded in a pre-LGM asphaltic deposit at Rancho La Brea, California. Palaeogeogr Palaeoclimatol Palaeoecol. 2020;537(109383):109383. 10.1016/j.palaeo.2019.109383

[9] Holden AR, Harris JM, Timm RM. Paleoecological and taphonomic implications of insect-damaged pleistocene vertebrate remains from Rancho La Brea, southern California. PLoS One. 2013;8(7):e67119. doi: 10.1371/journal.pone.0067119 23843988 PMC3700975

[10] Stock C, Harris JM. Rancho La Brea: a record of Pleistocene life in California. Los Angeles: Natural History Museum of Los Angeles County. Science Series. 1992; 37: 1–113.

[11] Mychajliw AM, Rice KA, Tewksbury LR, Southon JR, Lindsey EL. Exceptionally preserved asphaltic coprolites expand the spatiotemporal range of a North American paleoecological proxy. Sci Rep. 2020;10(1):5069. doi: 10.1038/s41598-020-61996-y 32193515 PMC7081288

[12] Brown C, Curd E, Friscia A. An actualistic experiment to determine skeletonization and disarticulation in the la Brea tar seeps. Palaios. 2017;32(3):119–24. 10.2110/palo.2016.074

[13] Lindsey EL, Seymour KL. “Tar Pits” of the western Neotropics: paleoecology, taphonomy, and mammalian biogeography. Natural History Museum of Los Angeles County Science Series. 2015; 42: 111–123.

[14] Noriega NL, Loyola Marymount University, Pitcher E, Cohen JE, Morado M, Yager A, et al. A stone’s throw through time: Taphonomic variation between pits at Rancho la Brea. In Geological Society of America; 2019. doi: 10.1130/abs/2019am-338840

[15] Simpson GG. The major features of evolution. New York: Columbia University Press. 1953.

[16] de Ricqlès A, de Buffrénil V. Bone histology, heterochronies and the return of tetrapods to life in water: where are we?, in: Mazin JM, de Buffrénil V. editors. Secondary adaptation of tetrapods to life in water. München: Verlag Dr. Friedrich Pfeil. 2001. p. 289–310. doi: 10.1038/35086500

[17] Laurin M, Canoville A, Germain D. Bone microanatomy and lifestyle: A descriptive approach. C R Palevol. 2011;10(5–6):381–402. 10.1016/j.crpv.2011.02.003

[18] Enlow DH, Brown SO. A comparative histological study of fossil and recent bone tissues. Part III. Texas Journal of Science. 1958; 10: 187–230.

[19] Francillon-Vieillot H, Buffrenil V, Castanet J, Géraudie J, Meunier FJ, Sire JY, et al. Microstructure and mineralization of vertebrate skeletal tissues, in: Carter JG, editors. Skeletal biomineralization: patterns, processes and evolutionary trends. New York: Van Nostrand Reinhold. 1990. p. 471–530.

[20] Klevezal GA. Recording structures of mammals. Determination of age and reconstruction of life history. Rotterdam: A.A. Balkema. 1996. doi: 10.1016/s0969-8043(96)00199-6

[21] Chinsamy-Turan A. The microstructure of dinosaur bone: deciphering biology with fine scale techniques. Baltimore: Johns Hopkins University Press. 2005.

[22] Chinsamy-Turan A. Forerunners of mammals: radiation, histology, biology. Bloomington: Indiana University Press. 2012.

[23] Padian K, Lamm ET. Bone histology of fossil tetrapods. Advancing methods, analysis, and interpretation. Berkeley and Los Angeles: University of California Press. 2013.

[24] Kolb C, Scheyer TM, Veitschegger K, Forasiepi AM, Amson E, Van der Geer AAE, et al. Mammalian bone palaeohistology: a survey and new data with emphasis on island forms. PeerJ. 2015;3(e1358):e1358. doi: 10.7717/peerj.1358 26528418 PMC4627922

[25] Stover SM, Pool RR, Martin B, Morgan JP. Histological features of the dorsal cortex of the third metacarpal bone mid-diaphysis during postnatal growth in thoroughbred horses. J Anat. 1992;181:455–469. 1304584 PMC1259699

[26] Martínez-Maza C, Alberdi MT, Nieto-Diaz M, Prado JL. Life-history traits of the Miocene Hipparion concudense (Spain) inferred from bone histological structure. PLoS One. 2014;9(8):e103708. doi: 10.1371/journal.pone.0103708 25098950 PMC4123897

[27] Nacarino-Meneses C, Chinsamy A, Mayda S, Kaya T, Erismis UC. Bone histology, palaeobiology, and early diagenetic history of extinct equids from Turkey. Quat Res. 2021;100:240–59. doi: 10.1017/qua.2020.87

[28] Nacarino-Meneses C, Chinsamy A. Mineralized-tissue histology reveals protracted life history in the Pliocene three-toed horse from Langebaanweg (South Africa). Zool. J. Linn. Soc. 2021; zlab037. 10.1093/zoolinnean/zlab037

[29] Hedges REM, Millard AR, Pike AWG. Measurements and relationships of diagenetic alteration of bone from three archaeological sites. J. Archaeol. Sci. 1995;22(2): 201–209. 10.1006/jasc.1995.0022

[30] Fernández-Jalvo Y, Andrews P, Pesquero MD, Smith C, Marín-Monfort D, Sánchez B, et al. Early bone diagenesis in temperate environments. Part I: surface features and histology. Palaeogeogr. Palaeoclimatol. Palaeoecol. 2010;288(1–4): 62–81. 10.1016/j.palaeo.2009.12.016

[31] Fernández-Jalvo Y, Andrews P. Atlas of taphonomic identifications. 1001 + images of fossil and recent mammal bone modification. Vertebrate Paleobiology and Paleoantropology Series. Dordrecht: Springer. 2016.

[32] Pesquero MD, Bell LS, Fernández-Jalvo Y. Skeletal modification by microorganisms and their environments. Hist. Biol. 2017;30(6): 882–893. 10.1080/08912963.2017.1371713

[33] Bell LS. Histotaphonomy, in: Crowder C and Stout S, editors. Bone histology: an anthropological perspective. CRC Press, Boca Raton. 2012. p. 241–254.

[34] Leidy J. Bones and teeth of horses from California and Oregon. Proc. Acad. Nat. Sci. Philadelphia. 1865.17(2):1–94.

[35] Stock C. Significance of abraded and weathered mammalian remains from Rancho La Brea. Bulletin of the Southern California Academy of Sciences. 1929;28:1–5.

[36] Feranec RS, Hadly EA, Paytan A. Stable isotopes reveal seasonal competition for resources between late Pleistocene bison (*Bison*) and horse (*Equus*) from Rancho La Brea, southern California. Palaeogeogr Palaeoclimatol Palaeoecol. 2009;271(1–2):153–60. 10.1016/j.palaeo.2008.10.005

[37] Fuller BT, Harris JM, Farrell AB, Takeuchi G, Southon JR. Sample preparation for radiocarbon dating and isotopic analysis of bone from Rancho La Brea, Los Angeles, California. La Brea and beyond: The paleontology of asphalt-preserved biotas, ed. Harris JM. Natural History Museum of Los Angeles County, Science Series. 2015;42:151–167.

[38] Cerda I, Pereyra M, Garrone M, Ponce D, Navarro T, González R, et al. A basic guide for sampling and preparation of extant and fossil bones for histological studies. Publ electrón Asoc Paleontol Argent [Internet]. 2020. 10.5710/peapa.07.04.2020.314

[39] Lamm ET.Preparation and Sectioning of Specimens, in: Padian K, Lamm ET, editors. Bone histology of fossil tetrapods: advancing methods, analysis and interpretation. Berkeley: University of California Press. 2013. p. 55–160.

[40] Padian K, de Boef Miara M, Larsson HCE, Wilson L, Bromage T. Research applications and integration, in: Padian K, Lamm ET, editors. Bone histology of fossil tetrapods. Advancing methods, analysis, and interpretation. Berkeley: University of California Press, 2013. p. 265–285.

[41] Garrone MC, Ortíz HO, Prado JL. Thin sections techniques in fossil remains of mammals impregnated with asphalt [abstract]. 2nd Palaeontological Virtual Congress. 2020.

[42] Schneider CA, Rasband WS, Eliceiri KW. NIH Image to ImageJ: 25 years of image analysis. Nat Methods. 2012;9(7):671–5. doi: 10.1038/nmeth.2089 22930834 PMC5554542

[43] de Ricqlès A, Meunier FJ, Castanet J, Francillon-Vieillot H. Comparative microstructure of bone, in: Hall BKeditor. Bone, bone matrix and bone specific products. Vol. 3. Boston: CRC Press. 1991. p. 1–78.

[44] Köhler M, Marín-Moratalla N, Jordana X, Aanes R. Seasonal bone growth and physiology in endotherms shed light on dinosaur physiology. Nature. 2012;487(7407):358–61. doi: 10.1038/nature11264 22763443

[45] Huttenlocker AK, Woodward HN, Hall BK. The biology of bone, in: Padian K, Lamm ETeditors. Bone histology of fossil tetrapods. Advancing methods, analysis, and interpretation. Berkeley: University of California Press. 2013. p. 13–34. doi: 10.1073/pnas.1302323110

[46] Woodward HN, Padian K, Lee AH. Skeletochronology. In: Padian K, Lamm ET, editors. Berkeley: University of California Press; 2013. p. 195–2015.

[47] Behrensmeyer AK. Taphonomic and ecologic information from bone weathering. Paleobiology. 1978;4(2):150–62. 10.1017/s0094837300005820

[48] Alcalá L., 1994. Macromamíferos neógenos de la fosa de Alfambra-Teruel. Teruel: Instituto de Estudios Turolenses y Museo Nacional de Ciencias Naturales (CSIC). 1994. p.554

[49] Lyman RL. Vertebrate taphonomy. Cambridge: Cambridge University Press. 1994.

[50] Hackett CJ. Microscopical focal destruction (tunnels) in exhumed human bones. Med Sci Law. 1981;21(4):243–65. doi: 10.1177/002580248102100403 7321807

[51] Jans MME. Microbial bioerosion of bone-a review. Wisshak M, Tapanila L, editors. Berlin: Springer-Verlag; 2008.

[52] Pfretzschner H.U., 2000. Microcracks and fossilization of Haversian bone. Neues Jahrbbuch für Geologie und Paläontologie—Abhandlungen 216, 413–432. 10.1127/njgpa/216/2000/413

[53] Pfretzschner H.U., 2004. Fossilization of Haversian bone in aquatic environments. C. R. Palevol. 3, 605–616. 10.1016/j.crpv.2004.07.006

[54] Pfretzschner HU, Tütken T. Rolling bones—Taphonomy of Jurassic dinosaur bones inferred from diagenetic microcracks and mineral infillings. Palaeogeogr Palaeoclimatol Palaeoecol. 2011;310:117–123. 10.1016/j.palaeo.2011.01.026

[55] Sander PM, Andrássy P. Lines of arrested growth and long bone histology in Pleistocene large mammals from Germany: What do they tell us about dinosaur physiology? Palaeontographica Abteilung A. 2006;277:143–159.

[56] Zedda M, Lepore G, Manca P, Chisu V, Farina V. Comparative bone histology of adult horses (Equus caballus) and cows (Bos taurus). Anat Histol Embryol. 2008;37(6):442–5. doi: 10.1111/j.1439-0264.2008.00878.x 18671686

[57] Zedda M, Sathe V, Chakraborty P, Palombo MR, Farina V. A first comparison of bone histomorphometry in extant domestic horses (Equus caballus) and a Pleistocene Indian wild horse (Equus namadicus). Integr Zool. 2020;15(6):448–60. doi: 10.1111/1749-4877.12444 32297705

[58] Nacarino-Meneses C, Jordana X, Köhler M. First approach to bone histology and skeletochronology of Equus hemionus. C R Palevol. 2016;15(1–2):267–77. 10.1016/j.crpv.2015.02.005

[59] Nacarino-Meneses C, Jordana X, Köhler M. Histological variability in the limb bones of the Asiatic wild ass and its significance for life history inferences. PeerJ. 2016;4(e2580):e2580. doi: 10.7717/peerj.2580 27761353 PMC5068390

[60] Nacarino-Meneses C, Köhler M. Limb bone histology records birth in mammals. PLoS One. 2018;13(6):e0198511. doi: 10.1371/journal.pone.0198511 29924818 PMC6010216

[61] Orlandi-Oliveras G, Nacarino-Meneses C, Koufos GD, Köhler M. Bone histology provides insights into the life history mechanisms underlying dwarfing in hipparionins. Sci Rep. 2018;8(1):17203. doi: 10.1038/s41598-018-35347-x 30464210 PMC6249282

[62] Nacarino-Meneses C, Orlandi-Oliveras G. The life history of European Middle Pleistocene equids: first insights from bone histology. Hist Biol. 2021;33(5):672–82. 10.1080/08912963.2019.1655011

[63] Cuijpers S, Lauwerier RCGM. Differentiating between bone fragments from horses and cattle: a histological identification method for archaeology. Environ Archaeol. 2008;13(2):165–79. 10.1179/174963108X343281

[64] Horner JR, de Ricqlès A, Padian K. Variation in dinosaur skeletochronology indicators: implications for age assessment and physiology. Paleobiology. 1999;25(3):295–304. 10.1017/S0094837300021308

[65] Enlow DH. Principles of bone remodeling: an account of post-natal growth and remodeling processes in long bones and the mandible. Springfield:Thomas. 1963, p.131.

[66] Enlow DH. A study of the post-natal growth and remodeling of bone. American Journal of Anatomy. 1962;110:79–101. 10.1002/aja.1001100202 13890322

[67] Marín-Moratalla N, Jordana X, Köhler M. Bone histology as an approach to providing data on certain key life history traits in mammals: Implications for conservation biology. Mamm Biol. 2013;78(6):422–9. 10.1016/j.mambio.2013.07.079

[68] Jordana X, Marín-Moratalla N, Moncunill-Solè B, Nacarino-Meneses C, Köhler M. Ontogenetic changes in the histological features of zonal bone tissue of ruminants: A quantitative approach. C R Palevol. 2016;15(1–2):255–66.

[69] Calderón T, DeMiguel D, Arnold W, Stalder G, Köhler M. Calibration of life history traits with epiphyseal closure, dental eruption and bone histology in captive and wild red deer. J Anat. 2019;235(2):205–16. doi: 10.1111/joa.13016 31148188 PMC6637702

[70] Amson E, Kolb C, Scheyer TM, Sánchez-Villagra MR. Growth and life history of Middle Miocene deer (Mammalia, Cervidae) based on bone histology. C R Palevol. 2015;14(8):637–45. 10.1016/j.crpv.2015.07.001

[71] Kolb C, Scheyer TM, Lister AM, Azorit C, de Vos J, Schlingemann MAJ, et al. Growth in fossil and extant deer and implications for body size and life history evolution. BMC Evol Biol. 2015;15(1):19. doi: 10.1186/s12862-015-0295-3 25887855 PMC4332446

[72] Shaw CA, Quinn JP. Rancho La Brea: a look at coastal southern California’s past. California Geology. 1986;39:123–133.

[73] Quinn JP. Rancho La Brea: Geologic setting, Late Quaternary depositional patterns and mode of fossil accumulation. In: Heaph EG, Lewis WL, editors. Santa Ana: South Coast Geological Society Annual Field Trip Guide Book; 1992. p. 221–232.

[74] Shaw CA, Kunitomi DS, Mulqueen SP, Hesson B. The history, geology and paleontology of the La Brea tar pits. In 2007. p. 1–9.

[75] Robert C. Late Quaternary variability of precipitation in Southern California and climatic implications: clay mineral evidence from the Santa Barbara Basin, ODP Site 893. Quat Sci Rev. 2004;23(9–10):1029–40. 10.1016/j.quascirev.2003.11.005

[76] Turner-Walker G, Jans M. Reconstructing taphonomic histories using histological analysis. Palaeogeogr Palaeoclimatol Palaeoecol. 2008;266(3–4):227–35. 10.1016/j.palaeo.2008.03.024

[77] Jans MME, Nielsen-Marsh CM, Smith CI, Collins MJ, Kars H. Characterisation of microbial attack on archaeological bone. J Archaeol Sci. 2004;31(1):87–95. 10.1016/j.jas.2003.07.007

[78] Jans MME Histological characterisation of diagenetic alteration of archaeological bone. Institute for Geo and Bioarchaeology, Vrije Universiteit, Amsterdam; 2005.

[79] Kim J-S, Crowley DE. Microbial diversity in natural asphalts of the Rancho La Brea Tar Pits. Appl Environ Microbiol. 2007;73(14):4579–91. doi: 10.1128/AEM.01372-06 17416692 PMC1932828

[80] Child AM. Microbial taphonomy of archaeological bone. Stud Conserv. 1995;40(1):19. 10.2307/1506608

[81] Turner-Walker G. The chemical and microbial degradation of bones and teeth. In: Ron Pinhasi R, Mays S, editors. Chichester: John Wiley & Sons; 2008. p. 1–25.

[82] Turner-Walker G. Early bioerosion in skeletal tissues: persistence through deep time. Neues Jahrb Geol Palaontol Abh. 2012;265(2):165–83. 10.1127/0077-7749/2012/0253

[83] Kendall C, Eriksen AMH, Kontopoulos I, Collins MJ, Turner-Walker G. Diagenesis of archaeological bone and tooth. Palaeogeogr Palaeoclimatol Palaeoecol. 2018;491:21–37. 10.1016/j.palaeo.2017.11.041

[84] Turner-Walker G. Light at the end of the tunnels? The origins of microbial bioerosion in mineralised collagen. Palaeogeogr Palaeoclimatol Palaeoecol. 2019;529:24–38. 10.1016/j.palaeo.2019.05.020

[85] Wedl C., 1864. Ueber einen im zahnbein und knochen keimenden pilz [About a fungus nascent in tooth and bone]. Mineral Biology Erdkunde 50,171–193.

[86] Roux W. Über eine im Knochen lebende Gruppe von Fadenpilzen (Mycelites ossifragus. Zeitschrift für Wissenschaftliche Zoologie. 1887;45:227–254.

[87] Bell LS, Skinner MF, Jones SJ. The speed of post mortem change to the human skeleton and its taphonomic significance. Forensic Sci Int. 1996;82(2):129–40. doi: 10.1016/0379-0738(96)01984-6 8885373

[88] Millard A. The deterioration of bone. In: Brothwell DR, Pollard AM, editors. Chichester: John Wiley & Sons; 2001. p. 637–647.

[89] Trueman CN, Martill DM. The long-term survival of bone: the role of bioerosion. Archaeometry. 2002;44(3):371–82. 10.1111/1475-4754.t01-1-00070

[90] Marchiafava V, Bonucci E, Ascenzi A. Fungal osteoclasia: a model of dead bone resorption. Calc Tis Res. 1974;14(1):195–210. 10.1007/bf02060295 4843788

[91] Fernandez-Jalvo Y, Sanchez-Chillon B, Andrews P, Fernandez-Lopez S, Alcala Martinez L. Morphological taphonomic transformations of fossil bones in continental environments, and repercussions on their chemical composition. Archaeometry. 2002;44(3):353–61. 10.1111/1475-4754.t01-1-00068

[92] Turner-Walker G, Nielsen-Marsh CM, Syversen U, Kars H, Collins MJ. Sub-micron spongiform porosity is the major ultra-structural alteration occurring in archaeological bone. Int J Osteoarchaeol. 2002;12(6):407–14. 10.1002/oa.642

[93] Curd E. Microbial community diversity, function, and succession in California’s Mediterranean habitats: Unpublished Ph. [Los Angeles]: University of California; 2016.

[94] Pfretzschner H-U. Collagen gelatinization: the key to understand early bone-diagenesis. Palaeontographica. 2006;278(1–6):135–48.135–148. 10.1127/pala/278/2006/135

[95] O’Brien FJ, Brennan O, Kennedy OD, Lee TC. Microcracks in cortical bone: how do they affect bone biology? Curr Osteoporos Rep. 2005;3(2):39–45. doi: 10.1007/s11914-005-0002-1 16036100

[96] Rogoz A, Sawlowicz Z, Wojtal P. Diagenetic history of woolly mammoth (Mammuthus primigenius) skeletal remains from the archaeological site Cracow spadzista street (b), southern Poland. Palaios. 2012;27(8):541–9. 10.2110/palo.2011.p11-115r

[97] Tomassini Rodrigo L., Miño-Boilini Ángel R., Zurita Alfredo E., Montalvo Claudia I., Cesaretti Nora, Modificaciones fosildiagenéticas en Toxodon platensis Owen, 1837 (Notoungulata, Toxodontidae) del Pleistoceno Tardío de la provincia de Corrientes, Argentina. Revista Mexicana de Ciencias Geológicas [Internet]. 2015;32(2):283–292. https://www.redalyc.org/articulo.oa?id=57240685008

