# Supplementary figures and images for: First osteohistological and histotaphonomic approach of Equus occidentalis Leidy, 1865 (Mammalia, Equidae) from the late Pleistocene of Rancho La Brea (California, USA)
Source: PLoS One. 2021 Dec 28;16(12):e0261915. doi: 10.1371/journal.pone.0261915 (PMC8714125; doi:10.1371/journal.pone.0261915)

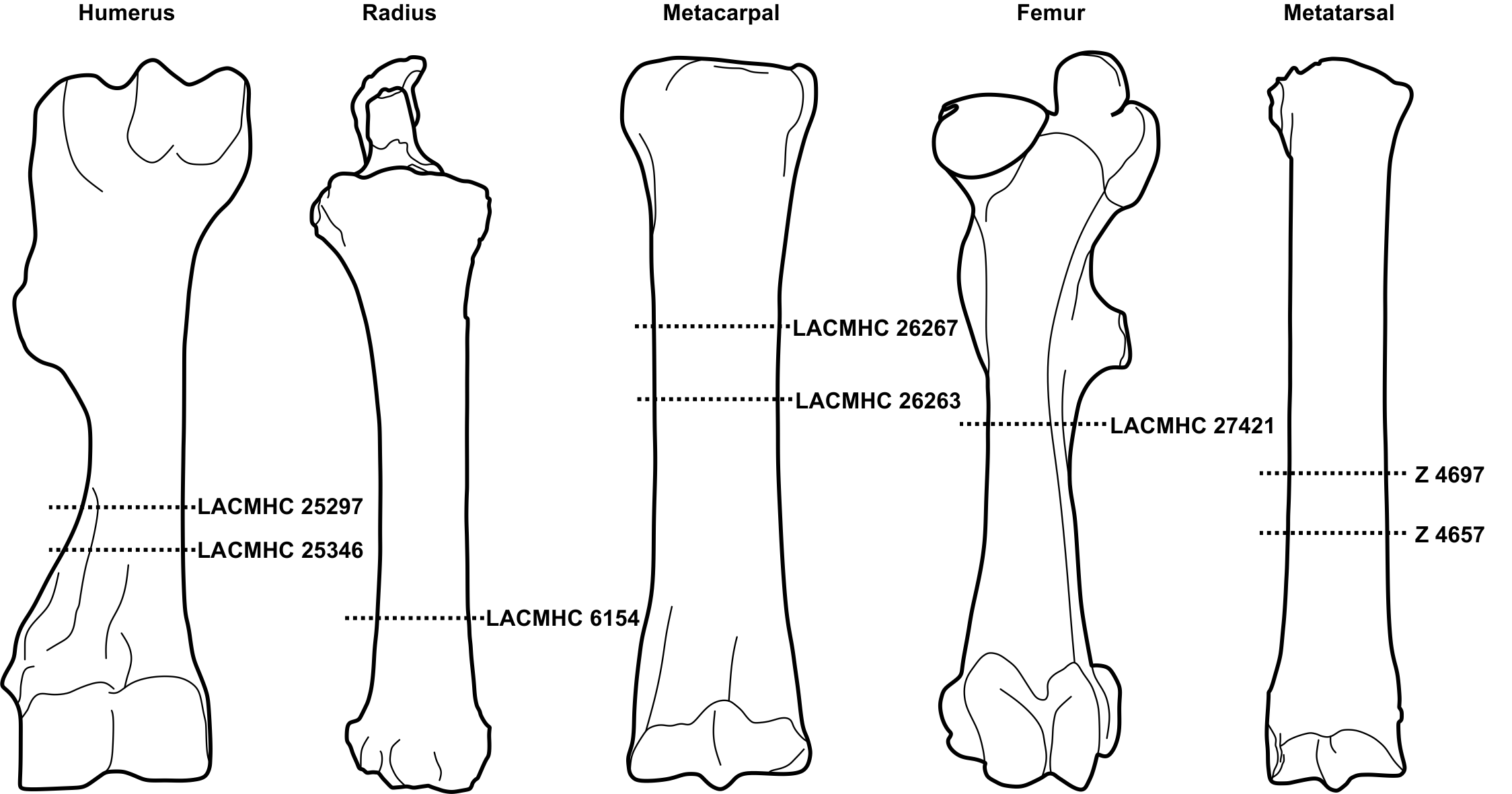

Supplement: S1 Fig — (TIF) [file pone.0261915.s001.tif]

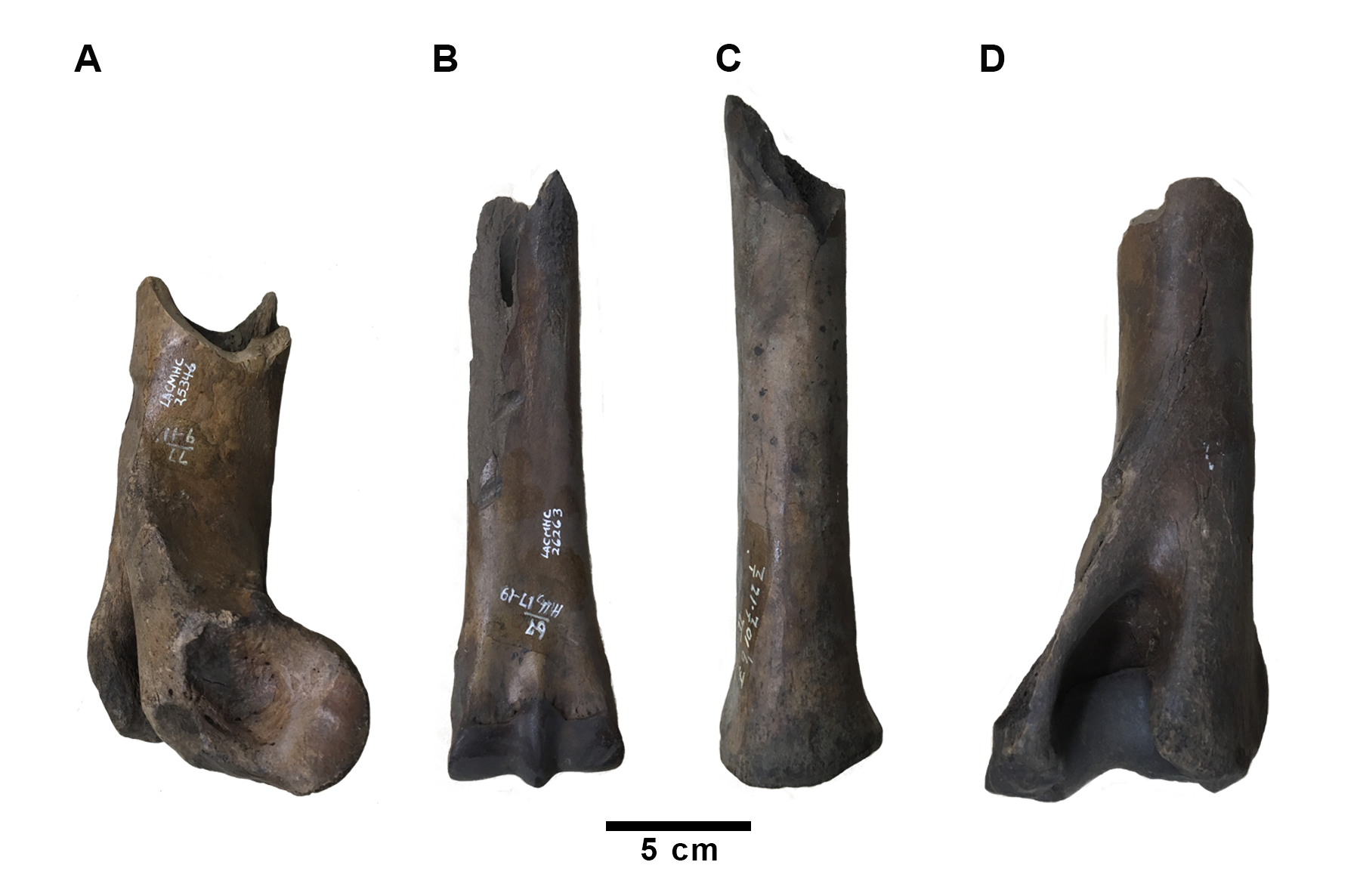

Supplement: S2 Fig — (A). Humerus LACMHC 25346, showing stage 0 of weathering and fossil-diagenetic fracture. (B). Mc-III LACMHC 26263, showing stage 0 of weathering and biostratinomic fracture. (C). Mt-III Z4697, showing stage 1 of weathering and fossil-diagenetic fracture. (D). Humerus LACMHC 25297, showing stage 1 of weathering and fossil-diagenetic fracture. Note that all the specimens show black color related to the impregnation with asphalt. The specimens do not show abrasion evidence. (TIF) [file pone.0261915.s002.tif]
